# A systematic review and meta-analysis of the prevalence of alcohol and other drug use and problematic use among people accessing mental health treatment in Australia

**DOI:** 10.1177/00048674251321272

**Published:** 2025-02-25

**Authors:** Christina Marel, Ewa Siedlecka, Jack Wilson, Sylvia Eugene Dit Rochesson, Daniel Chu, Alana Fisher, Katherine L Mills

**Affiliations:** 1 Matilda Centre for Research in Mental Health and Substance Use, The University of Sydney, Sydney, NSW, Australia; 2 School of Psychological Sciences, Macquarie University, Sydney, NSW, Australia

**Keywords:** Prevalence, substance use disorder, mental disorder, review, comorbidity

## Abstract

**Objective:**

Substance use and use disorders are elevated among people accessing mental health treatment, but the nature and patterns of use are unknown. The current study aimed to identify the prevalence of alcohol and other drug (AOD) use and problematic AOD use (i.e. hazardous, harmful, risky, misuse, abuse, dependence, disorder) in Australian mental health settings and conduct a meta-analysis of studies where sufficient data were available.

**Method:**

A systematic review of all papers published up to July 2023 identified 59 eligible studies reporting the prevalence of substance use, problematic use, and use disorders among people accessing mental health treatment in Australia. Overall, 55 studies provided sufficient data for a meta-analysis for past year use and problematic use of any AOD overall, alcohol, cannabis, tobacco, stimulants/amphetamines, and opioids.

**Results:**

Pooled prevalence estimates of past year use and problematic use among clients of mental health treatment settings varied (5%–58% and 7%–53%, respectively). Past year use and past year problematic use of tobacco were particularly prevalent (58% and 53%, respectively), as was cannabis (38% and 37%, respectively). Several key factors, including the type of mental health disorder, may explain some variation in prevalence estimates.

**Conclusion:**

The presence of co-occurring and problematic AOD use should be expected among a considerable proportion of clients of mental health treatment settings, and are a significant concern that services must be prepared to address. As such, screening and assessment of AOD use and use disorders should be part of routine clinical care, and clinicians should be familiar with evidence-based management and treatment strategies, including those that address tobacco.

## Introduction

Research conducted nationally and internationally has consistently demonstrated high rates of substance use and substance use disorders among those with mental health disorders (Jané-Llopis and Matytsina, 2006; McGrath et al., 2020). Data from the 2022 to 2023 NDSHS reported higher prevalence rates of past year substance use among people who had been diagnosed or treated for a mental health condition compared to those without, including daily smoking (15% vs 7%), alcohol (83% vs 80%), cannabis (20% vs 10%), ecstasy (3% vs 2%), and cocaine (7% vs 4%) use (Australian Institute of Health and Welfare, 2024a). These elevated prevalence rates are particularly concerning given there is evidence of a dose–response relationship between the use of some alcohol and other drug (AOD) and factors related to mental health, such as the onset of psychiatric symptoms, suicidal behaviours and psychological distress (Andreas et al., 2015; Conner and Bagge, 2019; Lagerberg et al., 2014).

Irrespective of the dose–response relationship between AOD use and more severe and complex mental health trajectories, even low use of AOD can have negative impacts, potentially exacerbating existing mental health symptoms or interacting with psychiatric or other medications (Hudson and Hudson, 2021; Mewton et al., 2020; Traccis et al., 2022; Twardowski et al., 2019; Wang et al., 2022). From a clinical perspective, co-occurring AOD and mental health disorders can present a diagnostic challenge, due to the similarities in symptom presentation (American Psychiatric Association, 2022), potentially interfering with the provision of accurate and timely intervention (Helseth et al., 2013; Kline and Mehler, 2006).

Despite evidence suggesting that AOD use and use disorders are elevated among people accessing mental health treatment (Prior et al., 2017; Sara et al., 2014), the nature and patterns of use are unclear. For example, it is unclear what types of AOD use and use disorders most commonly present to treatment, whether patterns reflect lifetime or current use, and – critically for mental health clinicians and services – what implications these have for mental health treatment. With previous research also demonstrating that most people who use AOD do not develop an AOD use disorder or dependence (Marel et al., 2019), it is vital to better understand the patterns and prevalence of both AOD use and use disorders in mental health treatment settings. This information would guide clinicians who work in such settings deliver informed, coordinated care, and better inform evidence-based decisions regarding service planning and delivery. Furthermore, the focus on Australian studies ensures conclusions are specific and relevant to Australian treatment settings, limiting the potential for national and cultural variation in substance use patterns.

The past few decades have seen an increasing body of research conducted in Australian mental health treatment settings capturing the prevalence of AOD use and use disorders. Much of the data, however, are clinically and methodologically diverse, which has likely limited attempts to conduct a rigorous review, synthesis and meta-analysis. The absence of such a synthesis makes it difficult for clinicians and service managers working in mental healthcare settings to apply key research findings to their practice. As such, the current systematic review aimed to (1) establish the prevalence of AOD use and problematic AOD use (i.e. hazardous, harmful, risky, misuse, abuse, dependence, use disorders) in Australian mental health settings; (2) examine what substances were most prevalent among those accessing mental health treatment, (3) determine whether patterns of use reflected current or lifetime prevalence and (4) where sufficient data were available, conduct a meta-analysis of studies, taking into account factors that may explain variance.

## Method

### Protocol and registration

The study protocol was registered on PROSPERO, the international prospective register of systematic reviews of the University of York (www.crd.york.ac.uk/prospero/; ID: CRD42021279070) and follows the recommendations of the Preferred Reporting Items for Systematic Reviews and Meta-analyses (PRISMA) guidelines (Page et al., 2021).

### Eligibility criteria

Studies were included if they met the following inclusion criteria: (a) published in English; (b) conducted in Australia; (c) reported prevalence rates of AOD use, AOD use disorder, abuse or dependence, or problematic/hazardous/risky use; (d) original data not reported elsewhere; (e) primary, secondary or tertiary mental health settings, including inpatient, outpatient, clinics, community-based organisations. Eligible study designs included controlled and uncontrolled (open) trials, pre-/post-studies, quasi-experimental studies, cohort and observational studies. Studies were excluded if they (a) were non-human studies; (b) included AOD use as part of eligibility criteria; (c) were not reporting original data (e.g. systematic reviews); (d) were conducted on people < 18 years; (e) any other healthcare setting (primary care, general practice, general hospital, accident/emergency); (f) AOD drug specialist settings and (g) non-treatment settings.

### Information sources and search

A systematic search was undertaken using the following databases: EMBASE, MEDLINE, PsycINFO and SCOPUS from inception to 26 July 2023. The following search term categories were used: substance use (AND) mental health (AND) comorbidity (AND) Australia. A full list of the search terms is available in Supplementary information Table 1.

### Study selection

After duplicates were removed, article titles and abstracts were independently double-screened for eligibility using COVIDENCE. Studies that received two ‘Yes’ ratings were selected for full-text screening, where final eligibility was determined and specific reasons for exclusion reported. Any conflicts in title/abstract rating or full-text review were resolved by discussion between reviewers.

### Data extraction procedure

The following data were extracted: sample size; sample characteristics (e.g. gender, age, education); year(s) of data collection; state/jurisdiction; type of AOD; type of mental health setting; primary mental health condition targeted during treatment; instruments used to generate prevalence data and prevalence estimates in percentages for any AOD use assessed.

### Outcomes

Due to the wide variation in the measurement of AOD use and use disorders, which included hazardous, harmful, risky, misuse, abuse, dependence, and use disorder, the term ‘problematic use’ was applied as an umbrella term to capture all harmful use of AOD. We defined ‘use’ as any reported use of AOD. We further stratified use by reference period, with ‘lifetime use’ defined as a reported history of AOD use, and ‘past year use’ as use in the last 12 months (including shorter timeframes, such as use at admission). Prevalence is reported for overall use, and within nine sub-categories of AOD (i.e. depressants, cannabinoids, tobacco, stimulants, opioids, hallucinogens, inhalants, polysubstance and ‘other’), with these sub-categories reporting individual AOD (e.g. alcohol) wherever possible.

### Quality of evidence and risk of bias in individual studies

The methodological quality of each study was evaluated by two reviewers using a modified version of Hoy et al.’s 10-item tool, which assesses internal and external validity of prevalence studies (Hoy et al., 2012). Each item was scored as low or high risk of bias, with an additional item assessing the overall bias rated as low, moderate or high. Studies that scored three or less low bias ratings on the first 10 items were given an overall rating of ‘high bias’; studies that scored between four and seven low bias ratings were given an overall rating of ‘moderate bias’; and studies that scored eight or more low bias ratings were given an overall rating of ‘low bias’.

### Statistical analysis

Given the range of estimated prevalence within extracted data, random-effects models and forest plots were used to pool effect sizes. Heterogeneity was measured using *I*^2^, which provides an overall estimate of variance due to heterogeneity, rather than incidence (Higgins et al., 2003). An *I*^2^ value of 0% suggests no observed heterogeneity, while values closer to 100% suggest increasing heterogeneity. Where there was heterogeneity (high *I*^2^), separate mixed-effects meta-regression models were conducted to investigate the impact of moderator variables. The following categorical moderators were included in separate subgroup analyses: primary mental disorder (psychosis, psychotic disorder or serious mental illness [psychosis or other serious mental illness] vs other, mixed or various), primary treatment setting (inpatient, mixed/outpatient vs outpatient), instrument used to assess substance use (file review vs other) and risk of bias (low vs other; Table 1). Meta-regression models were conducted for the following continuous variables: proportion of participants that were male and mean age of sample. Analysis was conducted using the *metafor* and *meta* packages in RStudio, R version 4.3.2 (Team, 2022). The package *metafor* was used to create models for meta-analysis, while the package *meta* was used to create forest plots. Significance was set at *p* < 0.05. For categories where there was an insufficient number of studies (*k* < 5) to conduct a meta-analysis, only prevalence ranges (minimum and maximum) are reported.

**Table 1.** Summary of predictor variables included in meta-analysis.

Predictor variables	Response options
Primary mental disorder	Psychosis, psychotic disorder or serious mental illness (psychosis or other serious mental illness) vs other, mixed or various
Primary treatment setting	Inpatient, mixed inpatient/outpatient vs outpatient
Proportion male	Continuous variable
Mean age	Continuous variable
Instrument used to assess AOD use	File review vs other
Risk of bias	Low vs other

## Results

**Figure 1.**
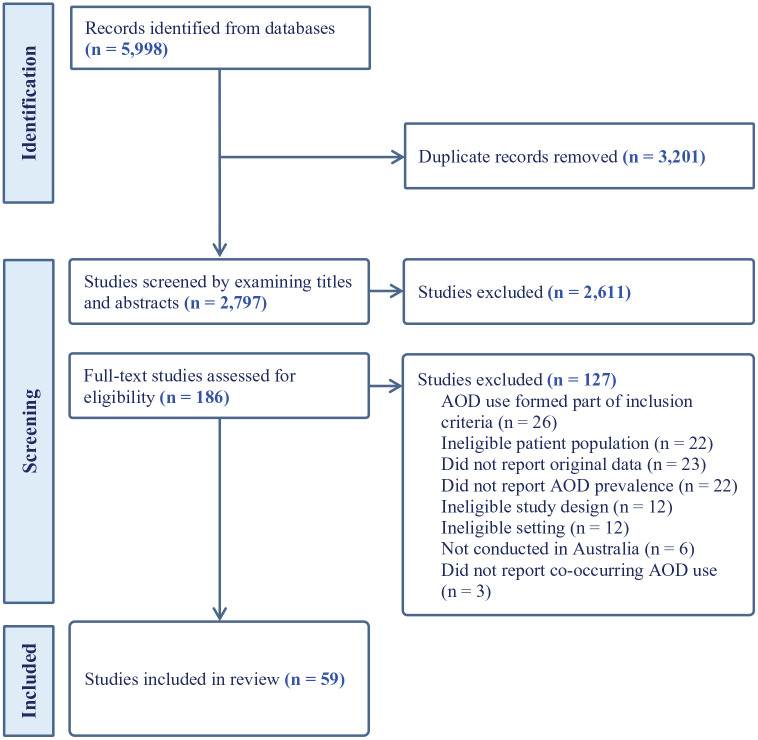
Flowchart for the study selection procedure.

The study selection criteria are shown in the PRISMA flowchart, Figure 1. After removing 3201 duplicates, there were 2797 identified records. Following screening of titles and abstracts, 186 full-text articles were assessed for eligibility. Overall, 59 studies met the inclusion criteria (Abrahams et al., 1970; Ash et al., 2003; Azraai et al., 2021; Bardell-Williams et al., 2019; Bartlem et al., 2015, 2018; Biddle et al., 2005; Branjerdporn et al., 2022; Charlson et al., 2021; Cleary et al., 2008; Conus et al., 2006; Davidson et al., 2001; Draper, 1994; Forbes et al., 2003; Fowler et al., 1998; Geffen et al., 2002; Gonda et al., 2012; Hambridge and Rosen, 1994; Hides et al., 2007; Hoolahan et al., 2006; Hunter et al., 2012; Iorfino et al., 2018; John et al., 2009, 2016; Kavanagh et al., 2011; Kent et al., 1995; Killackey et al., 2019; Korman et al., 2023; Lacey et al., 2007; Lai and Sitharthan, 2012; Lambert et al., 2005; Lee et al., 2010, 2013; Lin et al., 2015; Maccallum and Blaszczynski, 2002; Manning et al., 2017; Mellor et al., 2022; Morgan et al., 2006, 2012; Namrata and Oei, 2009; Nielssen et al., 2018; Ogloff et al., 2004, 2015; Parker et al., 2005; Perich et al., 2017; Reilly et al., 2019; Sara et al., 2014; Savilla et al., 2008; Searby et al., 2016; Smith et al., 2011; Stewart et al., 2019; Suomi et al., 2014; Tsoutsoulis et al., 2020; Vaddadi et al., 1997; Wade et al., 2005; Wye et al., 2010; Yee et al., 2022; Yellowlees and Kaushik, 1992; Zimmermann et al., 2012). Characteristics of these studies, including the overall bias rating for each, are presented in Table 2 of the supplementary materials. There were a sufficient number of studies to pool data on past 12-month any use and past 12-month problematic use of any AOD (7, 37); alcohol (11, 28); cannabis (13, 13); tobacco (13, 5), stimulants/amphetamines (12, 11) and opioids (5, 10). Meta-regressions from these studies are presented in Table 2.

**Table 2.** Meta-regression results of past 12-month use and problematic use of AOD, alcohol, cannabis, tobacco, stimulants and opioids.

Substance	Outcome	Moderator variable	Test statistics	*p*
Any AOD	Past 12-month use	Participants with a psychotic-related disorder vs those without	12.329	<0.001
		Treatment setting – inpatient only vs mixed/other	0.210	0.647
		Proportion of males	27.857	<0.001
		Mean age	13.760	<0.001
		Assessments – file review vs other	2.686	0.101
		Bias – low vs other	0.661	0.416
	Past 12-month problematic use	Participants with a psychotic-related disorder vs those without	30.218	0.001
		Treatment setting – inpatient only vs mixed/other	0.537	0.464
		Proportion of males	0.012	0.912
		Mean age	1.040	0.308
		Assessments – file review vs other	0.830	0.362
		Bias – low vs other	6.012	0.014
Alcohol	Past 12-month use	Participants with a psychotic-related disorder vs those without	0.059	0.808
		Treatment setting – inpatient only vs mixed/other	4.866	0.027
		Proportion of males	0.201	0.654
		Mean age	6.994	0.008
		Assessments – file review vs other	11.417	<0.001
		Bias – low vs other	4.044	0.044
	Past 12-month problematic use	Participants with a psychotic-related disorder vs those without	0.612	0.434
		Treatment setting – inpatient only vs mixed/other	0.225	0.635
		Proportion of males	0.301	0.583
		Mean age	0.006	0.939
		Assessments – file review vs other	1.683	0.195
		Bias – low vs other	0.138	0.711
Cannabis	Past 12-month use	Participants with a psychotic-related disorder vs those without	0.027	0.871
		Treatment setting – inpatient only vs mixed/other	5.985	0.014
		Proportion of males	5.377	0.020
		Mean age	1.345	0.246
		Assessments – file review vs other	0.601	0.438
		Bias – low vs other	0.008	0.931
	Past 12-month problematic use	Participants with a psychotic-related disorder vs those without	5.502	0.019
		Treatment setting – inpatient only vs mixed/other	0.202	0.653
		Proportion of males	0.026	0.872
		Mean age	9.925	0.002
		Assessments – file review vs other	0.593	0.441
		Bias – low vs other	0.503	0.478
**Tobacco**	Past 12-month use	Participants with a psychotic-related disorder vs those without	0.007	0.932
		Treatment setting – inpatient only vs mixed/other	0.504	0.478
		Proportion of males	0.581	0.446
		Mean age	2.665	0.103
		Assessments – file review vs other	0.383	0.536
		Bias – low vs other	0.428	0.513
	Past 12-month problematic use	Participants with a psychotic-related disorder vs those without	3.390	0.066
		Treatment setting – inpatient only vs mixed/other^a^	-	-
		Proportion of males	0.034	0.855
		Mean age	308.730	<0.001
		Assessments – file review vs other^a^	-	-
		Bias – low vs other	121.446	<0.001
Stimulants	Past 12-month use	Participants with a psychotic-related disorder vs those without	1.097	0.295
		Treatment setting – inpatient only vs mixed/other	4.470	0.034
		Proportion of males	23.085	<0.001
		Mean age	93.443	<0.001
		Assessments – other vs file review	0.314	0.575
		Bias – low vs other	3.244	0.072
	Past 12-month problematic use	Participants with a psychotic-related disorder vs those without	2.961	0.085
		Treatment setting – inpatient only vs mixed/other	8.745	0.003
		Proportion of males	0.082	0.774
		Mean age	0.039	0.844
		Assessments – other vs file review	2.566	0.109
		Bias – low vs other	5.374	0.020
Opioids	Past 12-month use	Participants with a psychotic-related disorder vs those without	0.015	0.903
		Treatment setting – inpatient only vs mixed/other	0.465	0.495
		Proportion of males	0.181	0.671
		Mean age	0.383	0.536
		Assessments – other vs file review	0.015	0.903
		Bias – low vs other	1.473	0.225
	Past 12-month problematic use	Participants with a psychotic-related disorder vs those without	6.079	0.014
		Treatment setting – inpatient only vs mixed/other	0.000	0.997
		Proportion of males	23.085	<0.001
		Mean age	0.065	0.799
		Assessments – other vs file review	0.687	0.407
		Bias – low vs other	0.017	0.897

a Unable to perform analysis, *N* = 0.

As noted, there was a significant variability in the prevalence data obtained across studies, including specific types of AOD reported (e.g. alcohol); AOD use as a broad category; sub-categories of AOD (e.g. stimulants) or a combination of these types of data. There were also significant differences in the measurement of problematic AOD use across studies (e.g. harmful use, hazardous use, abuse, dependence, use disorder), and variability in the reference period for assessing use (e.g. daily, weekly, 1-, 3-, 6-, 12-month, and lifetime estimates). For ease of interpretation, we have reported lifetime and 12-month use, where 12-month use also included shorter reference periods (e.g. 3-month use).

The risk of bias among the included studies was assessed as moderate, with samples unlikely to be representative of the mental health treatment seeking population, participants unlikely to be randomly selected, and studies unlikely to consider differences between responders and non-responders (Hoy et al., 2012).

### Co-occurring mental health disorders with any AOD use

#### Use of any substance

Nine studies reported on the prevalence of any AOD use among people attending mental health treatment services (Supplementary Table 3). Lifetime prevalence of any AOD use ranged between 50.0% and 100% for any substance (*k* = 3), while prevalence of any AOD use in the past 12 months ranged between 9.9% and 98.1% (*k* = 7).

**Figure 2.**
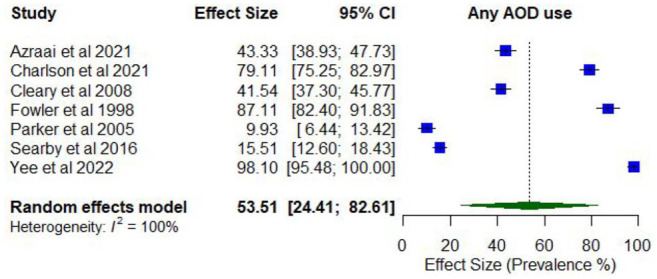
Prevalence of past 12-month AOD use.

Meta-analysis found a pooled proportion for past 12-month AOD use using random effects of 0.53 (95% CI = [0.24, 0.8]; *I*^2^ = 100%; Figure 2). The proportion of past 12-month AOD use differed according to whether participants were primarily experiencing psychosis or other serious mental illness (0.76; 95% CI = [0.52, 1.00]) compared to those experiencing other disorders (0.23; 95% CI = [0.05, 0.41]), and was higher among studies comprising higher proportions of males (0.75; 95% CI = [0.64, 0.83]) compared to lower proportions of males (0.43; 95% CI = [0.35, 0.51]), and with younger participants (lowest mean age 33.8 years; 0.67; 95% CI = [0.61, 0.73]) compared to older participants (highest mean age 78.2 years; 0.52; 95% CI = [0.40, 0.64]).

Overall, 41 studies reported on the prevalence of problematic AOD use (Supplementary Table 4). Lifetime prevalence of problematic use of any AOD ranged from 4.6% (abuse) to 77.7% (use disorder; *k* = 11), while the prevalence of problematic use in the last year ranged from 3.1% (abuse) to 88.3% (abuse; *k* = 41).

**Figure 3.**
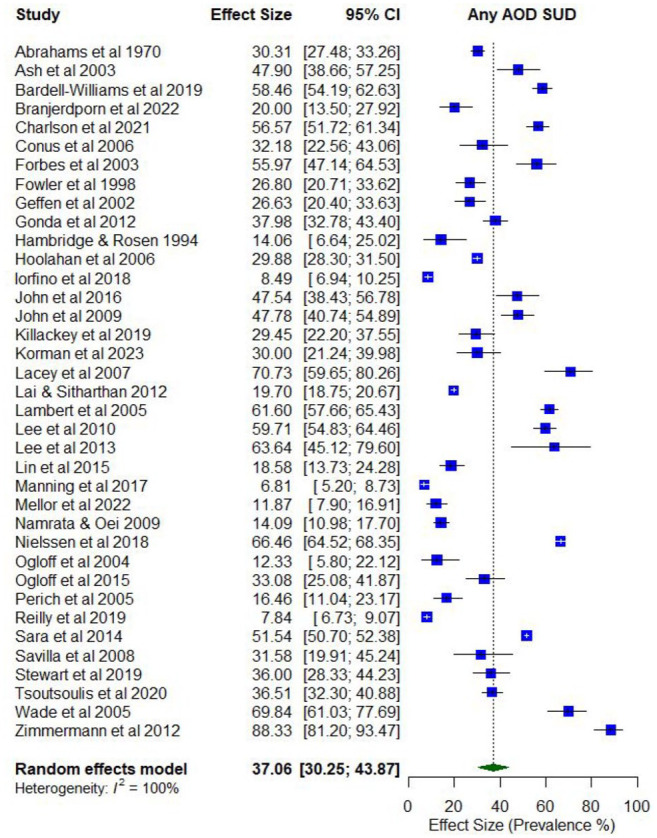
Prevalence of past 12-month problematic AOD use.

The pooled proportions for past 12-month problematic AOD use was 0.37 (95% CI = [0.30, 0.44]; *I*^2^ = 100%; Figure 3). Subgroup analysis illustrated that the proportion of past 12-month problematic AOD use differed according to whether participants were primarily experiencing psychosis or other serious mental illness (0.47; 95% CI = [0.42, 0.54]) compared to those experiencing other disorders (0.25; 95% CI = [0.19, 0.30]), and whether the risk of study bias was low (0.55; 95% CI = [0.40, 0.70]) compared to moderate/high (0.34; 95% CI = [0.27, 0.41]).

### Co-occurring mental health disorders and depressant use

The prevalence of co-occurring depressant use is shown in Supplementary Table 4. Where available, prevalence rates for specific types of depressants (i.e. alcohol, benzodiazepines, and sedatives, tranquilliser, hypnotics and barbiturates) have been included.

#### Alcohol use

Notably, 11 studies reported on the prevalence of any alcohol use. Lifetime prevalence of co-occurring alcohol use was assessed in one study only (98.9%), while past 12-month alcohol use ranged between 17.7% and 81.4% (*k* = 11).

**Figure 4.**
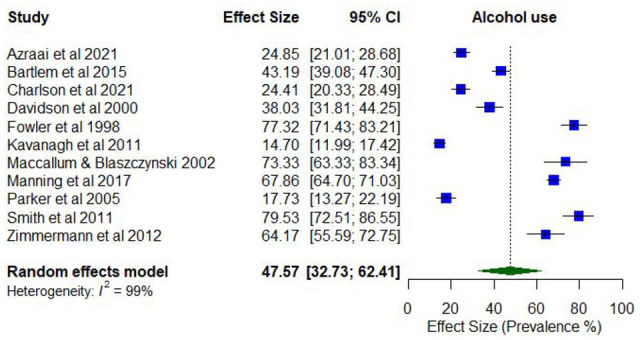
Prevalence of past 12-month alcohol use.

Meta-analysis found a pooled proportion for past 12-month alcohol use of 0.48 (95% CI = [0.33, 0.62]; *I*^2^ = 99%; Figure 4). The proportion of past 12-month alcohol use differed according to whether the treatment setting was inpatient only (0.28; 95% CI = [0.11, 0.44]) compared to mixed/outpatient settings (0.55; 95% CI = [0.37, 0.74]), whether prevalence was assessed via file review (0.22; 95% CI = [0.18, 0.27]) compared to other screening tools or measures of assessment (0.57; 95% CI = [0.37, 0.77]), and whether risk of study bias was low (0.64; 95% CI = [0.56, 0.73]) compared to medium/high (0.46; 95% CI = [0.30, 0.61]). The proportion of past 12-month alcohol use was also higher among studies with younger participants (lowest mean age 36.3 years; 0.68; 95% CI = [0.62, 0.74]) compared to older participants (highest mean age 44.8 years; 0.56; 95% CI = [0.49, 0.63]).

Overall, 33 studies reported on the prevalence of problematic alcohol use. Lifetime prevalence of problematic alcohol use ranged from 1.5% (abuse) to 54.6% (use disorder; *k* = 10). Prevalence estimates for problematic alcohol use in the last year ranged between 2.1% (abuse) and 70.5% (use problems; *k* = 28).

**Figure 5.**
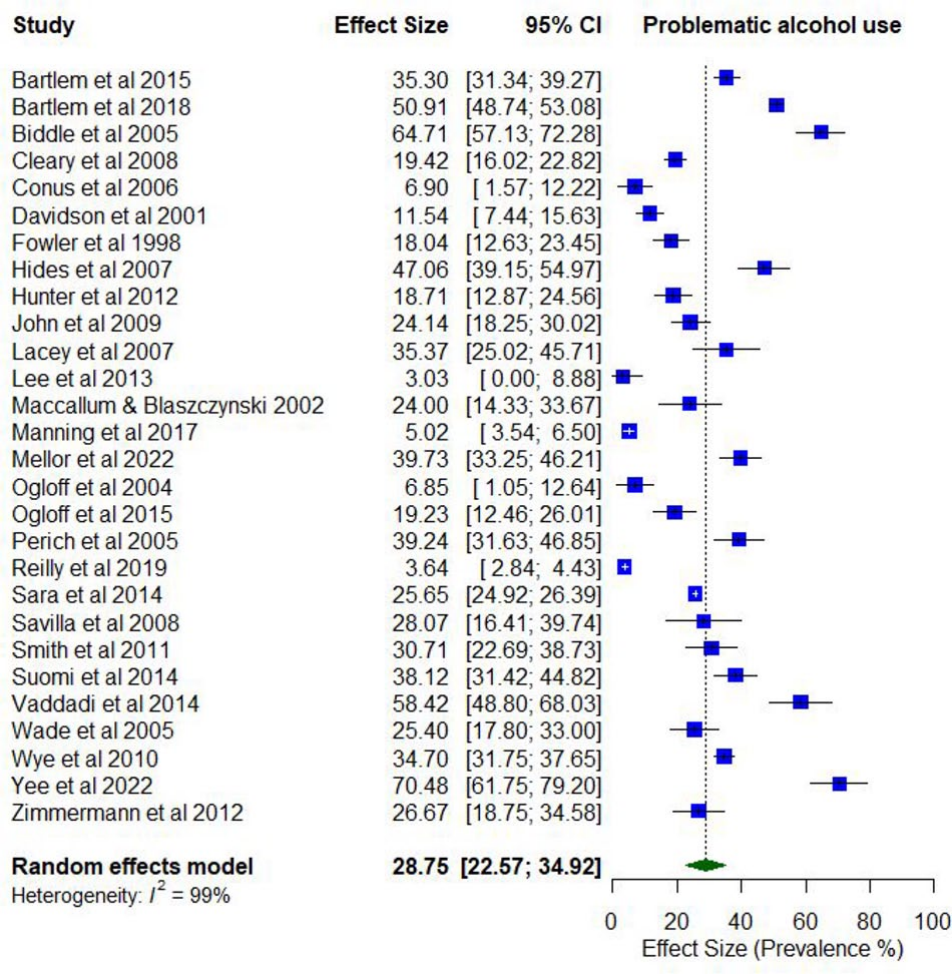
Prevalence of past 12-month problematic alcohol use.

The pooled proportion for past 12-month problematic use of alcohol use was 0.29 (95% CI = [0.23, 0.35]; *I*^2^ = 99%; Figure 5). No moderator variables were significantly associated with past 12-month problematic alcohol use.

#### Benzodiazepine use

Two studies examined prevalence of any benzodiazepine use, with lifetime prevalence of co-occurring benzodiazepine use assessed in only one study. Lifetime prevalence was 64.4% among a sample with schizophrenia, and prevalence of past 6-month benzodiazepine use was 10.6%.

Lifetime prevalence of problematic benzodiazepine use ranged from 1.0% (abuse) to 6.2% (dependence; *k* = 2). Prevalence estimates for past year problematic benzodiazepine use ranged from 1.5% (abuse, dependence) to 1.6% (use disorder; *k* = 2).

#### Sedative, tranquilliser, hypnotic or barbiturate use

Three studies reported past year prevalence of any sedative, tranquilliser, hypnotic or barbiturate use, with no studies examining lifetime prevalence. Estimates ranged from 9.1% for sedative use to 28.9% for barbiturate use (*k* = 3).

Six studies reported on the problematic use of sedatives, tranquillisers, hypnotics or barbiturates. Lifetime prevalence of problematic use of sedatives, tranquillisers, hypnotics, or barbiturates ranged from 6.9% for sedative and hypnotic use disorder to 20.0% for barbiturate abuse (*k* = 3). Past year prevalence for the problematic use of sedatives, tranquillisers, hypnotics, and barbiturates ranged between null and 19.5% (barbiturate dependence; *k* = 5), with higher prevalence for less severe use, but only for sedatives, tranquillisers and hypnotics.

### Co-occurring mental health disorders and cannabis use

Overall, 13 studies reported on the prevalence of any cannabis use (Supplementary Table 5). Lifetime prevalence of co-occurring cannabis use was examined in only one study, while cannabis use in the last year ranged between 13.3% and 92.2% (*k* = 13).

**Figure 6.**
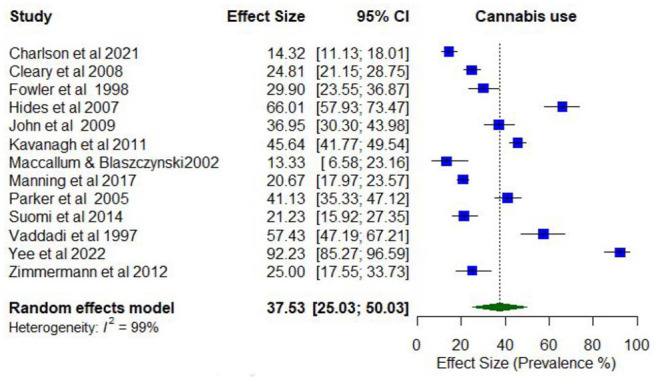
Prevalence of past 12-month cannabis use.

Meta-analysis found the pooled proportion for past 12-month cannabis use was 0.38 (95% CI = [0.26, 0.49]; *I*^2^ = 99%; Figure 6). The proportion of past 12-month cannabis use differed according to whether the treatment setting was inpatient only (0.57; 95% CI = [0.32, 0.82]) compared to mixed/outpatient settings (0.25; 95% CI = [0.19, 0.32]). The proportion of past 12-month cannabis use was also higher among studies with higher proportions of males (0.66; 95% CI = [0.51, 0.78]) compared to lower proportions of males (0.48; 95% CI = [0.38, 0.57]).

In total, 15 studies reported on the prevalence of problematic cannabis use. Lifetime prevalence of problematic cannabis use ranged from 7.7% (abuse) to 63.5% (use disorder; *k* = 6). Prevalence estimates for problematic cannabis use in the last year ranged between 4.1% (abuse) and 76.7% (daily use; *k* = 15).

**Figure 7.**
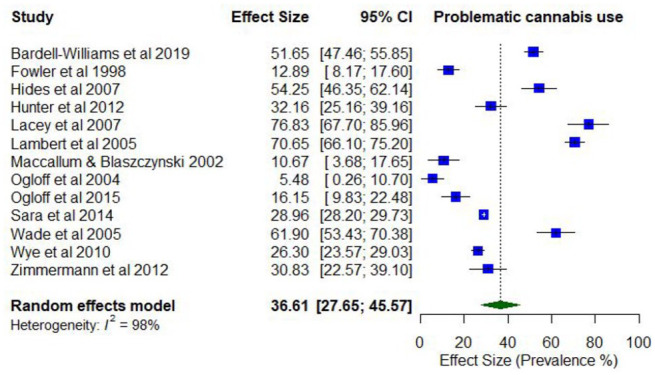
Prevalence of past 12-month problematic cannabis use.

The pooled proportion for past 12-month problematic cannabis use was 0.37 (95% CI = [0.28, 0.46]; *I*^2^ = 98%; Figure 7). The proportion of past 12-month problematic cannabis use differed according to whether participants were primarily experiencing psychosis or other serious mental illness (0.46; 95% CI = [0.32, 0.60]) compared to other disorders (0.22; 95% CI = [0.09, 0.35]). The proportion of past 12-month problematic cannabis use was also higher among studies with younger participants (lowest mean age 19.5 years; 0.63; 95% CI = [0.59, 0.66]) compared to older participants (highest mean age 40.2 years; 0.51; 95% CI = [0.48, 0.55]).

### Co-occurring mental health disorders and tobacco use

No studies reported lifetime prevalence of any tobacco use. However, 13 studies reported on the prevalence of any past year tobacco use, with estimates ranging from 47.0% to 76.3% (*k* = 13; Supplementary Table 6).

**Figure 8.**
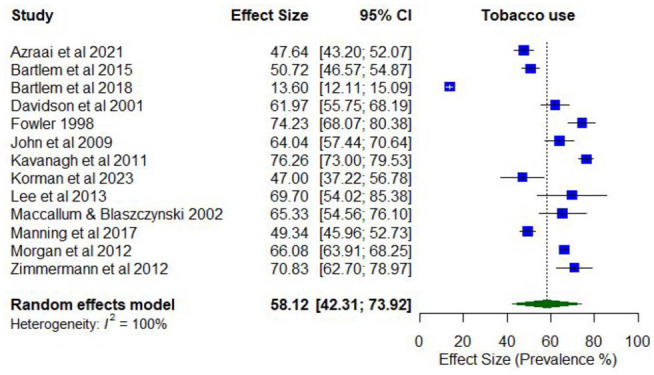
Prevalence of past 12-month tobacco use.

Meta-analysis found a pooled proportion for past 12-month tobacco use of 0.58 (95% CI = [0.42, 0.74]; *I*^2^ = 100%; Figure 8). No moderator variables were significantly associated with past 12-month tobacco use.

Only one study reported lifetime prevalence of problematic tobacco use (77% daily use). Five studies reported on the prevalence of past year problematic tobacco use, with estimates ranging between 16.7% (dependence) and 76.2% (daily use; *k* = 5).

**Figure 9.**
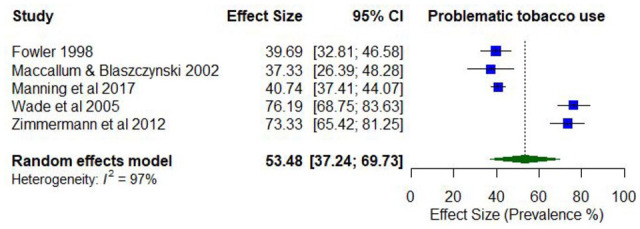
Prevalence of past 12-month problematic tobacco use.

The pooled proportion for past 12-month problematic tobacco use was 0.53 (95% CI = [0.37, 0.70]; *I*^2^ = 97%; Figure 9). The proportion of past 12-month problematic tobacco use differed according to whether the risk of study bias was low (0.75; 95% CI = [0.69, 0.80]) compared to moderate/high ratings (0.40; 95% CI = [0.37, 0.43]). The proportion of past 12-month problematic tobacco use was also higher among younger participants (lowest mean age 33.8 years; 0.71; 95% CI = [0.67, 0.75]) compared to the older participants (highest mean age 43.4 years; 0.52; 95% CI = [0.47, 0.57]).

### Co-occurring mental health disorders and stimulant use

Supplementary Table 5 illustrates the prevalence of co-occurring stimulant use. Where available, prevalence rates for specific types of stimulants (i.e. amphetamines, cocaine, and ecstasy) have been included. The prevalence of past year use and problematic use of stimulants and amphetamines was pooled for the purpose of meta-analysis.

#### Any stimulant/amphetamine use

No studies reported the lifetime prevalence of any stimulant use as a broad category. Two studies reported on the lifetime prevalence of any amphetamine use (34.0%–54.2%; *k* = 2). Two studies reported on the prevalence of any stimulant use in the past year, which ranged from 7.5% to 14.4% (*k* = 2; Supplementary Table 7). The prevalence of any amphetamine use in the past 12 months ranged from 1.3% to 91.3% (*k* = 10).

**Figure 10.**
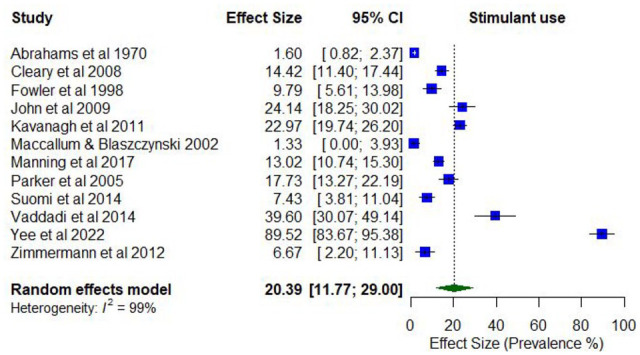
Prevalence of past 12-month stimulants/amphetamine use.

Meta-analysis found a pooled proportion for past 12-month stimulant/amphetamine use of 0.20 (95% CI = [0.12, 0.29]; *I*^2^ = 99%; Figure 10). The proportion of past 12-month stimulant/amphetamine use differed according to whether the treatment setting was inpatient only (0.42; 95% CI = [0.13, 0.70]) compared to mixed/outpatient settings (0.10; 95% CI = [0.05, 0.15]). The proportion of past 12-month stimulant use was also higher among studies with higher proportions of males (0.64; 95% CI = [0.54, 0.73]) compared to lower proportions of males (0.42; 95% CI = [0.54, 0.73]), and with participants who were younger (lowest mean age 33.8 years; 0.71; 95% CI = [0.67, 0.75]) compared to older (highest mean age 43.4 years; 0.52; 95% CI = [0.47, 0.57]).

Two studies reported lifetime prevalence of problematic stimulant use, which ranged from 8.2% (abuse) to 27.7% (use disorder). Three studies reported on the lifetime prevalence of problematic amphetamine use, with lifetime estimates ranging from 4.1% (abuse) to 22.9% (abuse).

The past year prevalence of problematic stimulant use ranged from 3.8% (use disorder) to 14.7% (use disorder). Past year prevalence of problematic amphetamine use ranged from 1.0% (abuse) to 41.7% (weekly injecting; *k* = 8).

**Figure 11.**
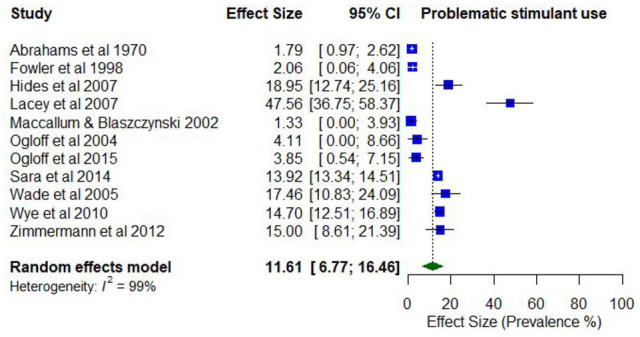
Prevalence of past 12-month problematic use of stimulant/amphetamines.

The pooled proportion for past 12-month problematic stimulant/amphetamine use was 0.12 (95% CI = [0.07, 0.16]; *I*^2^ = 99%; Figure 11). The proportion of past 12-month problematic stimulant/amphetamine use differed according to whether the treatment setting was inpatient only (0.15; 95% CI = [0.10, 0.20]) compared to mixed/outpatient (0.05; 95% CI = [0.02, 0.09]), and whether the risk of study bias was low (0.15; 95% CI = [0.13, 0.17]) compared to moderate/high bias (0.08; 95% CI = [0.04, 0.13]).

#### Cocaine use

Three studies reported on the prevalence of any cocaine use, with only one study reporting lifetime prevalence of any use (15.5%). Past year prevalence of any cocaine use ranged between 0% and 10.6% (*k* = 3).

The prevalence of problematic cocaine use was reported by four studies, with lifetime estimates ranging from 0 (abuse) to 4.1% (abuse; *k* = 4). Past year prevalence of problematic cocaine use ranged from 0 (abuse, dependence) to 4.2% (abuse; *k* = 4).

#### Ecstasy use

Three studies reported on the past year prevalence of any ecstasy use, with estimates ranging from 1.3% to 13.5%. No studies reported on lifetime prevalence of any ecstasy use or any problematic use.

### Co-occurring mental health disorders and opioid use

Where available, prevalence rates for specific types of opioids (i.e. heroin, analgesics, opiates and morphine) have been included (illustrated in Supplementary Table 8), but have been pooled across all opioids for the purpose of meta-analysis.

Only one study reporting on the lifetime prevalence of any opioid use (11.3% for non-prescribed opioids; 22.2% for prescribed opioids). No studies reported on the lifetime prevalence of any heroin use or any analgesic use.

Three studies reported on the past year prevalence of any opioid use, with estimates ranging between 2.6% (non-prescribed) and 6.7%. Two studies reported on the past year prevalence of any heroin use, with estimates ranging from 4.2% to 5.0%. Two studies reported on the past year prevalence of any analgesic use, with estimates ranging from 5.4% to 23.2%.

**Figure 12.**
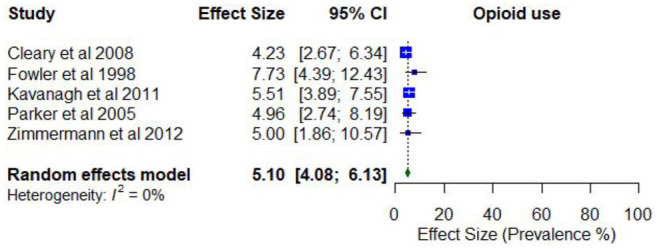
Prevalence of past 12-month opioid use.

Meta-analysis found a pooled proportion of past 12-month opioid use of 0.05 (95% CI = [0.04, 0.06]; *I*^2^ = 0%; Figure 12). No moderator variables were significantly associated with past 12-month opioid use.

Four studies reported lifetime prevalence of problematic use of any opioids, with estimates ranging from 0% (non-medical abuse of prescribed opioids) to 17.7% (use disorder). No studies reported on the lifetime prevalence of problematic analgesic use.

Past year prevalence of problematic use of any opioids ranged from 0% (use disorder, dependence) to 11.1% (use disorder; *k* = 8). Only one study reported on the past year prevalence of problematic heroin use (28.3%; weekly injecting), and one study reported past year prevalence of problematic morphine use (0.1%; dependence). Past year prevalence of problematic analgesic use was similarly reported in one study (16.5%; dependence).

**Figure 13.**
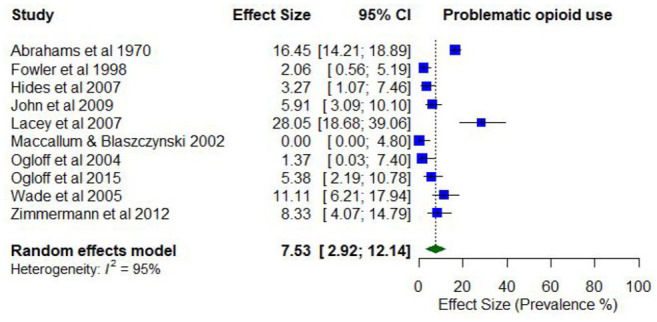
Prevalence of past 12-month problematic opioid use.

The pooled proportion of past 12-month problematic opioid use was 0.07 (95% CI = [0.03, 0.12]; *I*^2^ = 95%; Figure 13). The proportion of past 12-month problematic opioid use differed according to whether the treatment setting was inpatient only (0.09; 95% CI = [0.04, 0.15]) compared to mixed/outpatient settings (0.02; 95% CI = [0.00, 0.04]). The proportion of past 12-month problematic opioid use was also higher among studies with a higher proportion of males (0.64; 95% CI = [0.54, 0.73]) compared to a lower proportion of males (0.42; 95% CI = [0.36, 0.48]).

### Co-occurring mental health disorders and hallucinogen use

Three studies reported on the prevalence of any hallucinogen use, with lifetime estimates ranging between 37.1% and 37.5% (*k* = 2; Supplementary Table 9). The prevalence of any past year hallucinogen use ranged from 2.5% to 3.2% (*k* = 3).

Seven studies reported on the prevalence of problematic hallucinogen use, with lifetime estimates of problematic use ranging from 2.7% (dependence) to 20.0% (abuse; *k* = 5). The prevalence of past year problematic hallucinogen use ranged from 0% (abuse, dependence) to 12.7% (use disorder; *k* = 6).

### Co-occurring mental health disorders and inhalant use

Three studies reported on the prevalence of any inhalant use, with only one study estimating lifetime prevalence (18.5%; *k* = 1; Supplementary Table 10). Estimates of past year prevalence of any inhalant use ranged from 0.8% to 1.5% (*k* = 3).

Four studies reported on the prevalence of problematic inhalant use, with lifetime estimates ranging between 0.5% (abuse) and 3.6% (dependence; *k* = 2). The past year prevalence of problematic inhalant use ranged from 0% (dependence) to 3.3% (abuse; *k* = 4).

### Co-occurring mental health disorders and polysubstance use

Three studies reported on the past year prevalence of any polysubstance use, with estimates ranging from 2.7% to 22.8% (*k* = 3; Supplementary Table 11). No studies reported on the lifetime prevalence of any polysubstance use.

The prevalence of problematic polysubstance use, defined as the presence of at least two substance use disorders (Wade et al., 2005), was reported by three studies. Only one study estimated the lifetime prevalence of problematic polysubstance use disorder (42.1%; *k* = 1). Past year prevalence estimates of problematic polysubstance use ranged from 2.0% (use disorder) to 38.9% (use disorder; *k* = 3).

## Discussion

The prevalence of substance use and mental health conditions has remained persistently high in Australia and frequently co-occurs (Australian Bureau of Statistics, 2020–2022; Teesson et al., 2009). This review has demonstrated that while a significant proportion of people accessing mental health treatment also have co-occurring AOD use, there is substantial variability in the types of substances used, and patterns of use across included studies.

In relation to specific substances, prevalence estimates of lifetime use in the current review were higher than recently reported national prevalence estimates for alcohol (98.9% vs 85.1%), cannabis (66.0% vs 40.6%), amphetamines (34.0–54.2% vs 7.5%), cocaine (15.5% vs 13.5%), opioids (11.3–22.2% vs 5.7%), hallucinogens (37.1–37.5% vs 12.2%) and inhalants (18.5% vs 5.6%), respectively (Australian Institute of Health and Welfare, 2024b). Similarly, pooled prevalence estimates for past year use were higher in the current review compared to the general population in relation to cannabis (38% vs 11.5%), tobacco (58% vs 10.5%), stimulants/amphetamines (20% vs 1.0%) and opioids (5% vs 2.3%), respectively (Australian Institute of Health and Welfare, 2024b). Exceptions were observed for alcohol (48% in this review vs 76.9%), where estimates were lower in the current review in comparison to the general population.

As with any use, problematic AOD use showed similarly elevated patterns relative to the general population. In the current review, lifetime prevalence rates of problematic AOD use among those accessing mental health treatment were higher than lifetime estimates of use disorders among the general population for people using alcohol (1.5–54.6% in this review vs 21.0–23.3%), cannabis (7.7–63.5% in this review vs 5.5–6.9%), sedatives (1.0–20.0% in this review vs 0.4–0.8%), stimulants (1.5–27.7% in this review vs 2.8–3.9%) and opioids (0–17.7% in this review vs 0.6–1.1%) (Marel et al., 2019). As the comparative population data lack representativeness from populations who are marginalised and experience disadvantage associated with increased risk of AOD use, they are likely an underestimate of the actual prevalence. Nevertheless, the comparison demonstrates the higher prevalence of problematic AOD use among those accessing mental health treatment, suggesting that services should be prepared to address their presence among their clients.

Of particular concern in the current review are the high pooled prevalence rates of 12-month use and problematic tobacco use (58% and 53% respectively), which are substantially higher than the general population (10.5% and 8.3% respectively), who have shown decreases in smoking over the past few decades (Australian Institute of Health and Welfare, 2024b). Despite high rates of smoking among people with co-occurring AOD and mental health conditions being documented in previous research, and tobacco accounting for the highest mortality rates among people with comorbidity, there remains reluctance on the part of some clinicians to treat tobacco alongside clients’ mental illness or other AOD use, due to fears of exacerbating psychiatric symptoms, aggression or other AOD use (Cookson et al., 2014; Das and Prochaska, 2017; Jenkin et al., 2021). On the contrary, previous research suggests the opposite, with evidence-based smoking cessation strategies associated with improvements in depression (Shahab et al., 2014), anxiety (Hammett et al., 2019) and rates of smoking cessation (Das and Prochaska, 2017; Japuntich et al., 2020). There is a need to ensure effective and consistent implementation of smoking interventions in mental health services, with the inclusion of staff who smoke themselves (Jenkin et al., 2021).

Findings from the current review also suggest that rates of co-occurring AOD and mental health disorders may be lower in mental health settings compared to AOD treatment settings. A previous Australian review conducted among those attending AOD treatment found that the lowest prevalence estimate for mental health problems was 47% (Kingston et al., 2017), whereas the lowest prevalence estimate for problematic AOD use in the current review was 3%. These results suggest that problematic AOD use may be a stronger driver to seek treatment than mental health problems among people with co-occurring conditions.

### Factors that may influence prevalence estimates

It is important to note the substantial variation in prevalence estimates across the different substances, for both lifetime and past year use and problematic use. Findings from the meta-analysis suggest the variability may be explained, at least in part, by (1) the type of mental health disorder; (2) type of treatment setting; (3) method of assessing AOD use and (4) study setting, representativeness and demographic characteristics.

#### Type of mental health disorder

The 55 studies included in the meta-analysis comprised participants experiencing a range of mental health disorders, with just under half of studies (*n* = 24) focusing on people experiencing psychosis or other serious mental illness. The prevalence estimates of past 12-month AOD use, past 12-month problematic AOD use and past 12-month problematic cannabis use were significantly higher among samples with primarily psychosis or other serious mental illness compared to samples that were predominantly experiencing other disorders.

Notwithstanding these findings, due to the limitations of the data, the current review was unable to identify clear patterns of association between specific substances and specific mental health conditions, which is a fundamental tenant of the self-medication hypothesis; a theory which suggests that particular types of substances may be used by people experiencing co-occurring disorders due to the main action of the substance and its effect on specific psychological symptoms (e.g. alcohol, a depressant, used to calm anxiety-related symptoms) (Khantzian, 1997; Khantzian and Albanese, 2008). As such, the current review is unable to further clarify the nature of the complex relationships between mental health and AOD use.

### Type of treatment setting

The studies included in the meta-analysis comprised participants who were attending a range of treatment settings, and it is possible that particular patterns of AOD use may be more likely in some treatment settings than others, possibly due to other factors, such as type of substance being used by clients. However, it is not immediately apparent from the data that there are clear patterns in the prevalence estimates that can be accounted for by treatment setting. For example, those in inpatient settings had relatively low 12-month prevalence rates of alcohol use (28%) compared to those in mixed inpatient/outpatient or outpatient only settings (55%), but higher rates of 12-month cannabis use (57%), 12-month use and problematic use of stimulant/amphetamines (42% and 15%, respectively) and 12-month problematic use of opioids (9%) compared to those in mixed inpatient/outpatient only settings (vs 25% cannabis; 10% stimulant/amphetamine; 5% problematic stimulant/amphetamine; 2% opioids). It is possible that the higher rates of problematic use of stimulant/amphetamines and opioids in inpatient settings could be reflective of those with a more severe clinical profile, including more severe mental disorders and AOD use, but this is speculative.

#### Method of assessing AOD use

A range of methods were used to assess the prevalence of co-occurring AOD use, including diagnostic interviews, self-report questionnaires and the retrospective review of patient files. Findings from the current meta-analysis suggest that lower prevalence estimates of 12-month alcohol use were evident among studies that used retrospective file review (22%), compared to other assessments (57%). A previous review assessing the prevalence of mental health conditions among clients of AOD treatment services identified patterns between measurement method and prevalence estimates, with slightly higher estimates observed among studies that used self-report measures compared to clinical interviews (Kingston et al., 2017).

The higher estimates suggested that studies using screening tools may overestimate the ‘true prevalence’, implying that comorbidity exists where a full diagnostic interview may not necessarily lead to the same finding (Kingston et al., 2017). In the current meta-analysis, clinical and diagnostic interviews were combined with screening tools and assessment measures, to allow comparison with studies that had undertaken retrospective file reviews. It is unclear from the current analysis whether this variation may be due to retrospective file reviews underestimating the prevalence of 12-month alcohol use, or the possibility of their overestimation by other assessments. However, the lack of a significant association being identified for any other substance use in the meta-analysis suggests neither form of assessment may provide meaningful insight into interpreting variation.

#### Sample size, representativeness and demographic characteristics

The studies included in this review reflected a broad range of sample sizes (*n* = 33 to 13,624); studies with smaller samples were less likely to represent the target population. For example, the study with the smallest sample size (*n* = 33) reported some of the highest prevalence rates for past year problematic use of any AOD (63.6%), emphasising the importance of interpreting findings within the context of study limitations.

Differences in risk of bias may also explain some of the variation observed in the studies included in the current review, with more rigorous studies or those with lower risk of bias likely to produce more reliable findings. The current meta-analysis found higher prevalence estimates of past 12-month problematic AOD use (55%), alcohol use (64%), problematic stimulant/amphetamine use (15%), and problematic tobacco use (75%) among studies with low risk of bias compared to studies with moderate/high risk (34%; 46%; 8%; 40%, respectively).

In relation to demographic characteristics, meta-analysis findings demonstrated that prevalence estimates were higher among studies that comprised predominantly male participants for past 12-month AOD use, cannabis use and stimulant/amphetamine use. These findings generally align with patterns of use in the Australian general population, where men are more likely to use alcohol, tobacco, cannabis, cocaine, ecstasy, amphetamines, hallucinogens and inhalants compared to women (Australian Institute of Health and Welfare, 2024b).

Similarly, the prevalence of past 12-month AOD use, alcohol, cannabis and stimulant use, and problematic AOD, cannabis and tobacco use were higher in studies with younger participants. Again, the findings from this review are broadly consistent with patterns of use in the general Australian population, in which younger people are more likely to use AOD than older people (Australian Institute of Health and Welfare, 2024b).

Of the studies that examined the impact of socioeconomic status, most did not find any differences based on employment, type of housing or pension status. However, there was some evidence for higher AOD use among full-time workers compared to retired workers or pensioners (Suomi et al., 2014), and people who were homeless compared to those living in the family home (Cleary et al., 2008). These findings are consistent with previous research which has found elevated rates of AOD use and mental health disorders among people experiencing homelessness, compared to the Australian general population (Vallesi et al., 2021). However, there was not enough data on socioeconomic status to include in the meta-analysis and as such, these observations are purely descriptive.

### Implications for treatment and future research

The evidence in the current review suggests that co-occurring AOD use, including disorder-level and problematic use, is common among people accessing mental health treatment. As such, mental health treatment providers should expect the presence of AOD use, abuse, misuse, use disorders and dependence among their clients, and be prepared to provide appropriate treatment. It is therefore critical that mental health services routinely assess for the presence of AOD use, particularly tobacco use, among patients, regardless of their presenting mental health disorder.

It is also recommended that mental health services evaluate the way in which they provide and deliver services to ensure that co-occurring AOD use is not overlooked in their patients. Improving the capacity of the workforce is particularly important as the similarity in symptoms between AOD and mental health disorders can make distinguishing between substance-induced and independent disorders difficult. Recent focus on the need to improve the capacity of the AOD workforce to identify and respond to co-occurring mental health conditions has led to the development of evidence-based resources (e.g. Marel et al., 2016, 2021, 2022); however, research has identified a lack of similar resources and highlighted the need to support mental health workers in treating people with co-occurring AOD use (Fisher et al., 2014; Groenkjaer et al., 2017; Wheeler et al., 2014). Exemplifying this problem, an Australian study found that only 16% of people receiving mental health treatment were informed about their options for AOD treatment, which compared to 60% of people receiving treatment for AOD use, who were informed about their mental health treatment options (Barrett, 2019).

While diagnostic accuracy is critical for the delivery of appropriate and timely interventions, identifying co-occurring disorders can be challenging (American Psychiatric Association, 2022; Helseth et al., 2013). Despite this challenge, it is crucial that people experiencing mental health symptoms who are currently using substances, or who have a history of AOD use, are not automatically assumed to have a substance-induced disorder (Helseth et al., 2013), but are monitored and reassessed, both during treatment and after discharge. Ongoing assessment and symptom monitoring are crucial, as a considerable proportion (25–32%) of people who are diagnosed with substance-induced disorders are later diagnosed with independent mental disorders (Helseth et al., 2013; Murrie et al., 2020). Finding time to make complex diagnoses in busy mental health settings is an ongoing challenge, but has crucial implications for treatment and recovery.

The current review should be viewed in light of several limitations. First, the significant heterogeneity among the included studies made it difficult to draw firm conclusions regarding the association between specific study and participant characteristics, and prevalence rates. In addition, there was a significant variation in the sample sizes of studies included in this review. As studies with larger samples are generally more likely to represent the target population, the prevalence estimates from studies with small samples included in this review should be interpreted with caution.

Regarding implications for future research, there is a need for further high-quality, representative studies, that have the capacity to consider which factors contribute to the significant variability in prevalence rates reported in the current review. Future research should also focus on examining the impact of factors known to influence the prevalence of AOD use among those with mental disorders, including sex, age, ethnicity and socioeconomic status, and conduct additional analyses to examine the impact of these factors. Future studies could also systematically examine differences in co-occurring AOD use based on type of mental health condition and treatment setting, including comparisons between mental health and AOD services.

Findings from this review illustrate that approximately half of people accessing mental health services have used any AOD in the past year, with more than one-third having problematic use. Mental health workers and services should therefore expect to encounter AOD use, abuse, misuse, use disorder and dependence among their patients, and be prepared to provide appropriate and timely screening and assessment, diagnosis, and treatment that acknowledges and responds to AOD use in an appropriate and timely way.

## Supplemental Material

sj-pdf-1-anp-10.1177_00048674251321272 – Supplemental material for A systematic review and meta-analysis of the prevalence of alcohol and other drug use and problematic use among people accessing mental health treatment in Australia Supplemental material, sj-pdf-1-anp-10.1177_00048674251321272 for A systematic review and meta-analysis of the prevalence of alcohol and other drug use and problematic use among people accessing mental health treatment in Australia by Christina Marel, Ewa Siedlecka, Jack Wilson, Sylvia Eugene Dit Rochesson, Daniel Chu, Alana Fisher and Katherine L Mills in Australian & New Zealand Journal of Psychiatry
