# Supplementary material for: A systematic review and meta-analysis of the prevalence of alcohol and other drug use and problematic use among people accessing mental health treatment in Australia
Source: Aust N Z J Psychiatry. 2025 Feb 25;59(4):361–77. doi: 10.1177/00048674251321272 (PMC11924294; doi:10.1177/00048674251321272)
Supplement: sj-pdf-1-anp-10.1177_00048674251321272 – Supplemental material for A systematic review and meta-analysis of the prevalence of alcohol and other drug use and problematic use among people accessing mental health treatment in Australia [file sj-pdf-1-anp-10.1177_00048674251321272.pdf]

Supplementary Table 1: Database search terms

Database name	AOD-related terms	Mental health disorder-related terms	Prevalence-related terms	Limits
**Medline**	1. alcoholism/ or binge drinking/<br>2. exp Substance-Related Disorders/<br>3. ((abuse* or misuse* or dependenc* or addict* or disorder* or problem* or hazard* or harm* or risk*) adj4 (substance or sud or drug* or alcohol* or amphetamine* or cannabis or marijuana or cocaine or inhalant* or hallucinogen* or phencyclidine or heroin or morphine or opioid* or stimulant* or tobacco or sedative* or hypnotic or anxiolytic* or benzodiazepine or tobacco or nicotine or methamphetamine)).tw.<br>4. 1 or 2 or 3	5. exp Mental Disorders/<br>6. (depress* or dysthymi* or mental disorder* or mental illness* or psychological disorder* or psychological illness* or psychiatric disorder* psychiatric illness* or psychopatholog* or mood disorder* or affective disorder* or bipolar* or cychlothymi* or mania or manic or hypomania or anxiety disorder* or gad or panic disorder* or agoraphobi* or phobi* or obsessive-compulsive disorder or ocd or body dysmorphic disorder or bdd or hoarding disorder or trichotillomania or hair-pulling disorder or excoriation disorder or skin-picking disorder or post traumatic stress disorder or ptsd or acute stress disorder or adjustment disorder or psychotic or psychosis or schizo* or delusional disorder or dissociative disorder or conversion disorder or depersonali?ation disorder or dereali?ation disorder or psychosomati* or somati* or somatoform or eating disorder* or feeding disorder* or anorexi* or bulimi* or oppositional or defian* or conduct disorder* or pyromania or kleptomania or disruptive	8. prevalence/ or diagnosis, dual (psychiatry)/<br>9. exp Comorbidity/<br>10. (prevalen* or comorbid* or dual diagnos* or co-occur* or co-exist* or coexist* or concurrent).tw.<br>11. 8 or 9 or 10	12. Australia/<br>13. (australia*).tw.<br>14. 12 or 13<br>16. 4 and 7 and 11 and 14<br>17. limit 16 to (humans and English language)

		disorder or impulse control disorder or personality disorder* or attention deficit hyperactivity disorder or adhd or attention deficit disorder).tw.<br>7. 5 or 6		
**PsychINFO**	1. alcohol abuse/ or binge drinking/<br>2. exp alcoholism/<br>3. exp drug abuse<br>4. ((abuse* or misuse* or dependenc* or addict* or disorder* or problem* or hazard* or harm* or risk*) adj4 (substance or sud or drug* or alcohol* or amphetamine* or cannabis or marijuana or cocaine or inhalant* or hallucinogen* or phencyclidine or heroin or morphine or opioid* or stimulant* or tobacco or sedative* or hypnotic or anxiolytic* or benzodiazepine or tobacco or nicotine or methamphetamine)).tw.<br>5. 1 or 2 or 3 or 4	6. exp mental disorders/<br>7. (depress* or dysthymi* or mental disorder* or mental illness* or psychological disorder* or psychological illness* or psychiatric disorder* psychiatric illness* or psychopatholog* or mood disorder* or affective disorder* or bipolar* or cychlothymi* or mania or manic or hypomania or anxiety disorder* or gad or panic disorder* or agoraphobi* or phobi* or obsessive-compulsive disorder or ocd or body dysmorphic disorder or bdd or hoarding disorder or trichotillomania or hair-pulling disorder or excoriation disorder or skin-picking disorder or post traumatic stress disorder or ptsd or acute stress disorder or adjustment disorder or psychotic or psychosis or schizo* or delusional disorder or dissociative disorder or conversion disorder or depersonali?ation disorder or dereali?ation disorder or psychosomati* or somati* or somatoform or eating disorder* or feeding disorder* or anorexi* or bulimi* or oppositional or defian*	9. comorbidity/ or dual diagnosis/<br>10. (prevalen* or comorbid* or dual diagnos* or co-occur* or co-exist* or coexist* or concurrent).tw.<br>11. 9 or 10	12. (australia*).tw.<br>13. 5 and 8 and 11 and 12<br>13. limit 12 to (human and english language)

		or conduct disorder* or pyromania or kleptomania or disruptive disorder or impulse control disorder or personality disorder* or attention deficit hyperactivity disorder or adhd or attention deficit disorder).tw.<br>8. 6 or 7		
**EMBASE**	1. alcohol abuse/ or binge drinking/<br>2. exp alcoholism/<br>3. exp drug abuse/<br>4. substance abuse/<br>5. ((abuse* or misuse* or dependenc* or addict* or disorder* or problem* or hazard* or harm* or risk*) adj4 (substance or sud or drug* or alcohol* or amphetamine* or cannabis or marijuana or cocaine or inhalant* or hallucinogen* or phencyclidine or heroin or morphine or opioid* or stimulant* or tobacco or sedative* or hypnotic or anxiolytic* or benzodiazepine or tobacco or nicotine or methamphetamine)).tw.<br>6. 1 or 2 or 3 or 4 or 5	7. exp mental disease/<br>8. (depress* or dysthymi* or mental disorder* or mental illness* or psychological disorder* or psychological illness* or psychiatric disorder* psychiatric illness* or psychopatholog* or mood disorder* or affective disorder* or bipolar* or cychlothymi* or mania or manic or hypomania or anxiety disorder* or gad or panic disorder* or agoraphobi* or phobi* or obsessive-compulsive disorder or ocd or body dysmorphic disorder or bdd or hoarding disorder or trichotillomania or hair-pulling disorder or excoriation disorder or skin-picking disorder or post traumatic stress disorder or ptsd or acute stress disorder or adjustment disorder or psychotic or psychosis or schizo* or delusional disorder or dissociative disorder or conversion disorder or depersonali?ation disorder or dereali?ation disorder or psychosomati* or somati* or somatoform or eating disorder* or	10. prevalence/ or comorbidity/<br>11. (prevalen* or comorbid* or dual diagnos* or co-occur* or co-exist* or coexist* or concurrent).tw.<br>12. 10 or 11	13. australia/<br>14. (australia*).tw.<br>15. 13 or 14<br>16. 6 and 9 and 12 and 15<br>17. limit 16 to (human and english language)

		feeding disorder* or anorexi* or bulimi* or oppositional or defian* or conduct disorder* or pyromania or kleptomania or disruptive disorder or impulse control disorder or personality disorder* or attention deficit hyperactivity disorder or adhd or attention deficit disorder).tw.<br>9. 7 or 8		
**Scopus**	1. (abuse* OR misus* OR dependen* OR addict* OR disorder* OR problem* OR hazard* OR harm* OR risk* OR intoxicat*) W/4 (substance* OR sud OR drug* OR alcohol* OR amphetamine* OR cannabis OR marijuana OR cocaine OR inhalant* OR hallucinogen* OR phencyclidine OR heroin OR morphine OR opioid* OR stimulant* OR tobacco OR sedative* OR hypnotic* OR anxiolytic* OR benzodiazepine OR tobacco OR nicotine OR methamphetamine)<br>2. (alcoholi* OR "binge drink*" OR "substance related disorder*")<br>3. #1 or #2	4. (depress* OR dysthymi* OR "mental disorder*" OR "mental illness*" OR "psychological disorder*" OR "psychological illness*" OR "psychiatric disorder*" OR "psychiatric illness*" OR psychopatholog* OR "mood disorder*" OR "affective disorder*" OR bipolar* OR cychlothymi* OR mania OR manic OR hypomania OR "anxiety disorder*" OR gad OR "panic disorder*" OR agoraphobi* OR phobi* OR "obsessive-compulsive disorder" OR ocd OR "body dysmorphic disorder" OR bdd OR "hoarding disorder" OR trichotillomania OR "hair-pulling disorder" OR "excoriation disorder" OR "skin-picking disorder" OR "post traumatic stress disorder" OR ptsd OR "acute stress disorder" OR "adjustment disorder" OR psychotic OR psychosis OR schizo* OR "delusional disorder" OR "dissociative disorder" OR	5. (prevalen* OR comorbid* OR “dual diagnos*” OR co-occur* OR co-exist* OR coexist* OR concurrent)	6. (australia*)<br>7. #3 AND #4 AND #5 AND #6<br>8. (LIMIT-TO (LANGUAGE , "English")

		"conversion disorder" OR "depersonali?ation disorder" OR "dereali?ation disorder" OR psychosomati* OR somati* OR somatoform OR "eating disorder*" OR "feeding disorder*" OR anorexi* OR bulimi* OR oppositional OR defian* OR "conduct disorder*" OR pyromania OR kleptomania OR "disruptive disorder" OR "impulse control disorder" OR personality disorder* OR "attention deficit hyperactivity disorder" OR adhd OR "attention deficit disorder")		

Supplementary Table 2: Characteristics of included studies

Study	Treatment setting	Primary mental health disorder targeted	Year(s) of data collection	N	Sex, % male	Age, mean ± SD (years) or range	Australian jurisdiction	Instruments used to measure AOD prevalence	Risk of bias assessment summary[a]
Abrahams et al., (1970)	Inpatient and outpatient psychiatric hospital services	Not reported	Not reported	1003	37.9	Between 42.7 ± 16.2 and 44.3 ± 12.1	QLD	Developed own criteria	Moderate
Ash et al., (2003)	Acute psychiatric unit	Psychotic disorders	1997	119	69.7	35 ± 10	SA	File review	Moderate
Azraai et al., (2021)	Three hospitals with inpatient psychiatric services co-located with acute medical services and an on-site medical emergency team	Those being attended by the medical emergency team	2015-2020	487	50.9	44.8 ± 19.3	VIC	File review	Moderate
Bardell-Williams et al., (2019)	Early Psychosis Prevention and Intervention Centre	Psychotic disorders	2011 - 2013	544	59.7	19.5 ± 2.9	VIC	File review	Moderate
Bartlem et al., (2015)	Community mental health services	Depression	2011 - 2012	558	47.0	40.6 ± 15.1	NSW	Developed own criteria based on Australian national guidelines	Moderate
Bartlem et al., (2018)	Inpatient psychiatric facilities	Mood disorders	2012 - 2014	2075	55.8	41.5 ± 14.1	NSW	Developed own criteria based on Australian	Low

								national guidelines	
Biddle et al., (2005)	PTSD treatment programs	PTSD	Not reported	153	100.0	54.4 ± 4.9	VIC	AUDIT	Moderate
Branjerdporn et al., (2022)	Hospital mental health unit	General mental health illness	2020	130	53.1	21.3 ± 2.3	QLD	HoNOS	Low
Charlson et al., (2021)	Remote area mental health service	Psychotic disorders	1992 – 2015	426	65.6	Not reported	QLD	File review	Moderate
Cleary et al., (2008)	Acute inpatient psychiatric unit	Psychotic disorders	2006	520	59.0	38.3 ± 11.6	NSW	Developed own criteria based on Australian national guidelines	Low
Conus et al., (2006)	Early Psychosis Prevention and Intervention Centre	First-episode mania	1989 – 1997	87	55.2	22.1 ± 3.5	VIC	Royal Park Multidiagnostic Instrument for Psychosis	Moderate
Davidson et al., (2001)	Area mental health services	Various	Not reported	234	58.1	Mean not reported; range 18 – 65	VIC	National Health Survey, National Drug Strategy Household Survey, and Standard Tobacco Questions survey	Moderate
Draper (1994)	Public hospital psychiatric ward	Various	1984 – 1990	69	33.3	74; range 65 – 74	NSW	File review	Moderate
Forbes et al., (2003)	Inpatient and outpatient PTSD	PTSD	Not reported	134	Not reported	50.3 ± 3.77	VIC	AUDIT, MMPI-2, Addiction Admission Scale,	Moderate

	treatment program							MacAndrew Alcoholism–Revised content scale	
Fowler et al., (1998)	Community mental health clinics	Schizophrenia	Not reported	194	72.7	36.3; range 18 – 60	NSW	SCID-R	Moderate
Geffen et al., (2002)	Large psychiatric units in general hospitals	Psychotic disorders	1998 – 1999	184	58.7	38.8 ± 1.2	QLD	File review	Moderate
Gonda et al., (2012)	Inpatient psychiatric rehabilitation facility	Psychotic disorders	2003 – 2008	337	50.4	Between 33.4 ±10.3 and 33.7 ± 9.2	NSW	File review	Moderate
Hambridge & Rosen (1994)	Mobile community management team	Serious mental illness	1988 – 1992	64	64.1	44.3; range 19 – 74	NSW	BPRS, LSP	Moderate
Hides et al., (2007)	Acute psychiatric wards	Various	1998 – 2000	153	71.9	30.0 ± 11.5	QLD	AUDIT, section L of the CIDI, SDS, urine screen	Low
Hoolahan et al., (2006)	Community mental health centres; acute and psychiatric hospitals	Not reported	2001 – 2004	3209	47.9	Mean not reported; range 17 – 64	NSW	HONOS	Low
Hunter et al., (2012)	Rural and remote area mental health service	Psychotic disorders	2010	171	72.5	Mean not reported; range 17 – 68	QLD	File review	Low
Iorfino et al., (2018)	Youth mental health clinics	Various	2005 – 2018	1143	43.0	18.8 ± 3.8	NSW	File review	Moderate

John et al., (2009)	Public psychiatric service	Psychotic disorders	2005 – 2006	203	61.1	Between 39.2 ± 12.3 and 40.0 ± 11.5	WA	Unspecified	Moderate
John et al., (2016)	Public, tertiary care, inpatient psychiatric rehabilitation facility	Psychotic disorders	2010 – 2015	122	67.2	31.7 ± 8.8	WA	File review	Moderate
Kavanagh et al., (2011)	High security psychiatric unit; acute inpatient admissions to hospitals	Psychotic disorders	Not reported	653	70.4	Between 24.8 ± 5.3 and 32.6 ± 11.1	QLD	AUDIT, CIDI, SDS, DrugCheck Problem List, DrugCheck Recent Substance Use	Moderate
Kent et al., (1995)	Community mental health centres; an acute inpatient/ outpatient psychiatric hospital	Psychotic disorders	1989 – 1991	50	48.0	Between 33.2 ± 11 and 34.1 ± 9.9	SA	DAST, MAST	Moderate
Killackey et al., (2019)	Early Psychosis Prevention and Intervention Centre	Psychotic disorders	2009 – 2011	146	69.2	20.4 ± 2.4	VIC	SCID-R, OTI	Moderate
Korman et al., (2023)	Community care unit	Psychosis	2011 – 2017	100	64.0	34.0 ± 10.6	QLD	File review	Moderate
Lacey et al., (2007)	Inpatient unit of a public hospital	Serious mental illness	2002 – 2003	82	Not reported	30; SD not reported	VIC	Risk Behaviour Questionnaire	Moderate
Lai & Sitharthan (2012)	Psychiatric and general public and private hospitals	Various	2006 – 2007	6716	60.8	Mean not reported; range 0 – 80+	NSW	File review	Moderate

Lambert et al., (2005)	Early Psychosis Prevention and Intervention Centre	Psychotic disorders	1998 – 2000	625	67.0	21.6 ± 3.4	VIC	File review	Moderate
Lee et al., (2010)	Assertive community outreach	Various	2006 – 2007	417	0.0 – 71.1	Between 31.7 – 40.3; range 17 – 75	VIC	File review	Moderate
Lee et al., (2013)	Psychiatric inpatient unit	Various	2007 – 2011	33	90.9	31.8 ± 8.0	VIC	File review	Moderate
Lin et al., (2015)	Specialist clinic for young people at ultra-high risk for psychosis	Various	1993 – 2006	226	44.7	18.6 ± 3.3	VIC	SCID	Moderate
MacCallum & Blaszczynski., (2002)	Outpatient gambling treatment service	Gambling disorder	Not reported	75	64.0	37.7 ± 10.6	NSW	CIDI	Moderate
Manning et al., (2017)	Outpatient mental health services	Various	2015 – 2016	837	50.9	38 ± 13	VIC	AUDIT-C, HSI, SDS, Drug Use Screen	Moderate
Mellor et al., (2022)	Inpatient mental health treatment facility	PTSD	2019-2020	219	97.3	62.5 ± 14.6	QLD	File review	Moderate
Morgan et al., (2006)	Inpatient and outpatient psychiatric services	Psychotic disorders	Not reported	687	60.8	Between 37 ± 12 and 40 ± 11	ACT, QLD, VIC, WA	Diagnostic interview for psychosis	Low
Morgan et al., (2012)	Mental health services	Psychotic disorders	2010 – 2011	1825	59.6	Mean not reported; range 18 – 64	NSW, QLD, SA, VIC, WA	Diagnostic interview for psychosis, AUDIT, CAGE, FTND	Moderate

Namrata & Oei, (2009)	Outpatient gambling treatment service	Gambling disorder	2000 – 2002	440	50.9	42.7 ± 12.2	QLD	Developed own questions	Moderate
Nielssen et al., (2018)	Mental health clinics at homeless hostels	Psychotic disorders	2008 – 2016	2388	93.5	Between 42.3 ± 11.1 and 42.3 ± 12.8	NSW	File review	Moderate
Ogloff et al., (2004)	Forensic psychiatric hospital	Various	Not reported	73	83.2	40.2 ± 14.5	VIC	SCID-IV	Moderate
Ogloff et al., (2015)	Forensic mental health service	Various	Not reported	130	100.0	33.2 ± 10.0	VIC	SCID-IV	Moderate
Parker et al., (2005)	Mood disorders unit clinic	Mood disorders	Not reported	282	26.2	Mean not reported, range 32 – 37	NSW	File review	High
Perich et al., (2017)	Specialist outpatient bipolar disorders clinic	Bipolar disorders	Not reported	158	0.0	37.9 ± 13.6	NSW	SCID-I, CIDI	Moderate
Reilly et al., (2019)	Acute mental health inpatient unit	Various	2009 – 2014	2118	58.9	Mean not reported, range 18 – 65	QLD	File review	Moderate
Sara et al., (2014)	Mental health units in public hospitals	Schizophrenia	2000 - 2011	13624	66.9	32.6 ± 8.8	NSW	File review	Low
Savilla et al., (2008)	Community mental health service	Schizophrenia	Not reported	57	75.4	36.1 ± 9.1	SA	MINI, QOL	Moderate
Searby et al., (2016)	Older adult psychiatry service	Mood disorders, dementia	2012 - 2014	593	44.5	78.2 ± 9.8	VIC	File review	Moderate
Smith et al., (2011)	Gambling treatment services	Gambling disorder	2008	127	54.3	43.1 ± 12.7	SA	AUDIT	Moderate

Stewart et al., (2019)	Inpatient mental health units in five public hospitals	Psychotic disorders	2013 – 2016	150	63.3	40.6 ± 11.1	NSW	File review	Low
Suomi et al., (2014)	Gambling treatment services	Gambling disorder	Not reported	212	50.5	43.4 ± 13.1	SA, TAS, VIC	AUDIT-C, ASSIST, smoking single item question	Moderate
Tsoutsoulis et al., (2020)	Nonacute inpatient mental health rehabilitation unit in a tertiary care hospital	Various	Not reported	504	67.9	43.4 ± 0.82	NSW	File review	Moderate
Vaddadi et al., (1997)	General hospital psychiatric unit	Various	Not reported	101	60	Mean not reported, range 18 – 54	VIC	Unspecified	Moderate
Wade et al., (2005)	Early Psychosis Prevention and Intervention Centre; and two area mental health services	Psychotic disorders	1997; 2001	126	70.6	21.5 ± 3.5	VIC	CUAD	Low
Wye et al., (2010)	Psychiatric inpatient hospital service	Suicidal ideation, dementia	2005 – 2006	1000	Not reported	Mean, SD, range not reported	NSW	File review	Moderate
Yee et al., (2022)	Prison mental health services	Psychotic disorders	2015 – 2016	105	100	33.8 ± 9.3	NSW	SCID	Moderate
Yellowlees & Kaushik, (1992)	Psychiatric hospital service; prison medical service; private psychological practice	Various	1986 - 1990	707	50	37, range 4 – 87	NSW	File review	Moderate

Zimmermann et al., (2012)	Community-based mental health organisation	Serious mental illness	2009	120	56.7	Mean not reported, range 16 – 30	NSW, SA, VIC, WA	ASSIST, Tobacco Questionnaire	Low

[a]Summary item from a modified version of Hoy et al.'s 10-item tool, which assesses the internal and external validity of prevalence studies {Hoy, 2012 #58}. Studies that scored ≤3 low bias ratings on the first 10 items were given an overall rating of 'high bias'; studies that scored 4-7 low bias ratings were given an overall rating of 'moderate bias'; and studies that scored ≥8 low bias ratings were given an overall rating of 'low bias'.

ASSIST = Alcohol, Smoking, and Substance Involvement Screening Test; AUDIT = Alcohol Use Disorders Identification Test; BPRS = Brief Psychiatric Rating Scale; CIDI = Composite International Diagnostic Interview; CO = carbon monoxide; CUAD = Chemical Use, Abuse, and Dependence Scale; DAST = Drug Abuse Screening Test; DSM = Diagnostic and Statistical Manual of Mental Disorders; DUDIT = Drug Use Disorders Identification Test; FTND = Fagerström Test for Nicotine Dependence; HoNOS = Health of the Nation Outcome Scales; HSI = Heaviness of Smoking Index; ICD = International Classification of Diseases; LSP = Life Skills Profile; MAST = Michigan Alcohol Screening Test; MINI = MINI International Neuropsychiatric Interview; MMPI = Minnesota Multiphasic Personality Inventory; NHMRC = National Health and Medical Research Council; OTI = Opiate Treatment Index; PTSD = post-traumatic stress disorder; QOL = Quality Of Life; SCID = Structured Clinical Interview for DSM; SDS = Severity of Dependence Scale; TLFB = Time Line Follow Back.

Supplementary Table 3: Prevalence of any co-occurring AOD use among people accessing mental health treatment

Study	Any use		Problematic use[$]	
	**Lifetime**	**Past 12 months[%]**	**Lifetime**	**Past 12 months[%]**
**Any AOD use %, *n***				
Abrahams et al., (1970)				30.3 (dependence), *304*[§]
Ash et al., (2003)				47.9 (abuse), *57*[§]
Azraai et al., (2021)		43.3, *211*[§]		
Bardell-Williams et al., (2019)				58.5 (abuse)*, 318*[§]
Branjerdporn et al., (2022)				20.0 (abuse), 26[§]
Charlson et al., (2021)		79.1, *337*[§]		56.6 (use disorder), *241*[§]
Cleary et al., (2008)		41.5, *216*[†]		
Conus et al., (2006)				32.2 (use disorder), *28*[§]
Draper (1994)			13.0 (abuse), *9*	
Forbes et al., (2003)				56.0 (use disorder), *75*[§]
Fowler et al., (1998)	100.0, *194*	87.1, *169*[‡]	4.6 (abuse) – 55.2 (dependence), *9–107*	3.1 (abuse) – 23.7 (dependence), *6–46*[‡]
Geffen et al., (2002)				26.6 (use disorder), *49*[§]
Gonda et al., (2012)				38.0 (use disorder), *128*[§]
Hambridge & Rosen, (1994)				14.1 (problematic use), *9*[§]

Hoolahan et al., (2006)				29.9 (problematic use), *959*[§]
Iorfino et al., (2018)				8.5 (use disorder), *97*[§]
John et al., (2009)				47.8 (misuse), *97*[§]
John et al., (2016)				47.5 (use disorder), *58*[§]
Kent et al., (1995)			30.0 (abuse), *15*	
Killackey et al., (2019)				29.5 (use disorder), *43*[†]
Korman et al., (2023)				30.0 (use disorder), *30*[§]
Lacey et al., (2007)	50.0, *41*			70.7 (misuse), *58*[§]
Lai & Sitharthan, (2012)				19.7 (use disorder), *1323*[§]
Lambert et al., (2005)			74.1 (use disorder), *463*	61.6 (use disorder), *385*[§§]
Lee et al., (2010)				8.2 (use disorder) – 51.6 (misuse), *34 – 215*[§]
Lee et al., (2013)			18.2 (use disorder), *6*	63.6 (use disorder), *21*[§]
Lin et al., (2015)				21.9 (use disorder), *42*[§]
Manning et al., (2017)			12.8 (use disorder), *107*	6.8 (use disorder), *57*[§]
Mellor et al., (2022)				11.9 (use disorder), *26*[§]
Morgan et al., (2012)			54.5 (use disorder), *995*	
Namrata & Oei, (2009)				14.1 (dependence), *62*[§]
Nielssen et al., (2018)				66.1 (use disorder), *1,587*[§]
Ogloff et al., (2004)			73.9 (abuse disorder), *54*	12.3 (abuse or dependence disorder), *9* [§]

Ogloff et al., (2015)			77.7 (use disorder), *101*	33.1 (use disorder), *43*[§]
Parker et al., (2005)		9.9, *28*[§]		
Perich et al., (2017)				7.6 (abuse) – 8.9 (dependence), *12-14*[#]
Reilly et al., (2019)				7.8 (use disorder) 166[§]
Sara et al., (2014)				43.7 (use disorder) – 51.5 (use disorder), *5,952 – 7,022*[§]
Savilla et al., (2008)				31.6 (abuse), *18*[§]
Searby et al., (2016)		15.5, *92*[§]		
Stewart et al., (2019)				36.0 (use disorder), *54*[§]
Tsoutsoulis et al., (2020)				36.5 (use disorder), *184*[#]
Wade et al., (2005)			71.4 (use disorder), *90*	69.8 (use disorder), *88*[#]
Yee et al., (2022)		98.1, *103*[§]		
Yellowlees & Kaushik (1992)			10.0 (abuse), *71*	
Zimmermann et al., (2012)	97.5, *117*			31.7 (dependence) – 88.3 (abuse), *38 – 106*[$$]

[$] *Includes problematic use up to disorder level: harmful, misuse, abuse, dependence, or disorder level*

[%] *Includes assessment periods up to 12 months prior to treatment entry: current, 1-, 3-, 6-, or 12-months*

[§] *Assessment period "current"; i.e., at admission to treatment or undefined*

[†] *Assessment period 1 month prior to treatment entry*

[‡] *Assessment period 6 months prior to treatment entry*

[#] *Assessment period 12 months prior to treatment entry*

[$$] *Assessment period 3 months prior to treatment entry*

Supplementary Table 4: Prevalence of co-occurring depressant use among people accessing mental health treatment

Study	Any use		Problematic use[$]	
	**Lifetime**	**Past 12 months[%]**	**Lifetime**	**Past 12 months[%]**
**Alcohol %, *n***				
Azraai et al, (2021)		24.6, *121*[§]		
Bartlem et al., (2015)		43.2, *241*[†]		35.3 (chronic risk) – 40.3 (short-term risk), *197–225*[†]
Bartlem et al., (2018)				50.9 (hazardous use), *1,037*[§]
Biddle et al., (2005)				65.0 (use disorder), *99*[#]
Charlson et al., (2021)		24.2, *103*[§]		
Cleary et al., (2008)				19.4 (harmful use), *101*[‡]
Conus et al., (2006)				6.9 (use disorder), *6*[§]
Davidson et al., (2001)		38.0, *89*[#]		11.5 (harmful), *27*[#]
Draper (1994)			18.8 (abuse), *13*	
Fowler et al., (1998)	98.9, *192*	77.4, *150*[%%]	1.5 (abuse) – 46.9 (dependence), *3–91*	2.1 (abuse) – 16.0 (dependence), *4–31*[%%]
Hides et al., (2007)				47.1 (risky use), *72*[#]
Hunter et al., (2012)				18.7 (clinical impact), *32*[§]
John et al., (2009)				24.1 (misuse), *49*[§]
Kavanagh et al., (2011)		81.4, *96*[%%]		
Kent et al., (1995)			20.0 (abuse) – 34.3 (alcoholism), *10–12*	
Lacey et al., (2007)				35.0 (risky use), *29*[§]
Lee et al., (2013)				3.3 (use disorder), *1*[§]
Maccallum & Blaszczynski, (2002)		73.3, *55*[#]		8.0 (dependence) – 16.0 (abuse), *6–12* [#]

Manning et al., (2017)		67.9, *568*[#]	9.1 (use disorder), *76*	5.0 (use disorder) – 37.5 (hazardous use), *42–314*[#]
Mellor et al., (2022)				39.7 (use disorder), *87*[§]
Morgan et al., (2006)			23.6 (abuse), *162*	
Morgan et al., (2012)			50.5 (use disorder), *922*	
Ogloff et al., (2004)			21.9 (abuse) – 31.5 (dependence), *16–23*	6.8 (abuse), *5*[§]
Ogloff et al., (2015)			54.6 (use disorder), *71*	19.2 (use disorder), *25*[§]
Parker et al., (2005)		17.7, *50*[#]		
Perich et al., (2017)				17.7 (abuse) – 21.5 (dependence), *28–34*[§]
Reilly et al., (2019)				3.6 (use disorder), *77*[§]
Sara et al., (2014)				25.7 (use disorder), *3,495*[§]
Savilla et al., (2008)				28.1 (abuse), *16*[§]
Smith, et al., (2011)		79.5, *101*[#]		14.2 (dependence) – 16.5 (risky or harmful),*18–21*[#]
Suomi et al., (2014)				38.1 (abuse), *77*[%%]
Vaddadi et al., (1997)				58.4 (heavy use), *59*[§]
Wade et al., (2005)			27.8 (use disorder), *35*	25.4 (use disorder), *32*[#]
Wye et al., (2010)				34.7 (use disorder), *347*[§]
Yee et al., (2022)				70.5 (use problems), *74* [§]
Yellowlees & Kaushik, (1992)			32.0 (use disorder), *226*	
Zimmermann et al., (2012)		64.2, *77*[§§]		6.7 (dependence) – 26.7 (abuse), *8–32*[§§]
**Benzodiazepines %, *n***				
Fowler et al., (1998)	64.4, *125*	10.6, *21*[%%]	1.0 (abuse) – 6.2 (dependence), *2–12*	1.5 (abuse, dependence), *3*[%%]

Wade et al., (2005)		2.4 (use disorder), *3*	1.6 (use disorder), *2*[#]
**Sedative, tranquiliser, hypnotic and barbiturate %, *n***			
Abrahams et al., (1970)	23.2 – 28.9, *233–290*[§]		4.5 (dependence) – 19.5 (dependence), *45–196*[§]
Kent et al., (1995)		20.0 (abuse), *7*	
Manning et al., (2017)	9.1, *76*[#]		
Ogloff et al., (2004)		5.5 (abuse) – 9.6 (dependence), *4–7*	2.7 (abuse), *2*[§]
Ogloff et al., (2015)		6.9 (use disorder), *9*	0 (use disorder), *0*[§]
Yellowlees & Kaushik, (1992)		16.0 (abuse), *113*	
Zimmermann et al., (2012)	15.0, *18*[§§]		2.5 (dependence) – 15.0 (abuse), *3–18*[§§]

[$] *Includes problematic use up to disorder level: heavy consumption, harmful use, risky use, problematic use, hazardous use, chronic use, misuse, abuse, dependence, or disorder level*

[%] *Includes assessment periods up to 12 months prior to treatment entry: current, 1-, 3-, 6-, or 12-months*

[§] *Assessment period "current"; i.e., at admission to treatment or undefined*

[†] *Assessment period 1 month prior to treatment entry*

[‡] *National Health and Medical Research Council (2009). Australian Guidelines to Reduce Health Risks from Drinking Alcohol. Canberra, ACT: Commonwealth of Australia*

[#] *Assessment period 12 months prior to treatment entry*

[$$] *National Health and Medical Research Council. (2001). Australian alcohol guidelines: Health risks and benefits. Canberra: NHMRC.*

[%%] *Assessment period 6 months prior to treatment entry*

[§§] *Assessment period 3 months prior to treatment entry*

Supplementary Table 5: Prevalence of co-occurring cannabis use among people accessing mental health treatment

Study	Any use		Problematic use[$]	
	**Lifetime**	**Past 12 months**[%]	**Lifetime**	**Past 12 months**[%]
**Cannabis %, *n***				
Bardell-Williams et al., (2019)				51.7 (abuse), *281*[§]
Charlson et al., (2021)		14.3, *61*[§]		
Cleary et al., (2008)		24.8, *129*[†]		
Fowler et al., (1998)	66.0, *128*	29.9, *58*[‡]	7.7 (abuse) – 28.3 (dependence), *15–55*	4.1 (abuse) – 8.8 (dependence), *8–17*[‡]
Hides et al., (2007)		66.0, *101*[#]		54.2 (dependence), *83*[#]
Hunter et al., (2012)				32.2 (clinical impact), *55*[§]
John et al., (2009)		36.9, *75*[§]		
Kavanagh et al., (2011)		45.6, *298*[$$]		
Kent et al., (1995)			31.4 (abuse), *11*	
Lacey et al., (2007)				76.7 (daily use), *63*[§]
Lambert et al., (2005)				43.5 (use disorder), *272*[$$]
Maccallum & Blaszczynski, (2002)		13.3, *10*[#]		5.3 (abuse), 5.3 (dependence), *4, 4*[#]
Manning et al., (2017)		20.7, *173*[#]		
Morgan et al., (2006)			25.3 (abuse), *174*	
Ogloff et al., (2004)			11.0 (abuse) – 34.2 (dependence), *8–25*	5.5 (abuse), *4*[§]
Ogloff et al., (2015)			51.6 (use disorder), *65*	16.7 (use disorder), *21*[§]
Parker et al., (2005)		41.1, *116*[#]		
Sara et al., (2014)				29.0 (use disorder), *3,946*[§]
Suomi et al., (2014)		21.3, *45*[$$]		
Vaddadi et al., (1997)		57.4, *58*[§]		

Wade et al., (2005)		63.5 (use disorder), *80*	61.9 (use disorder), *78*[#]
Wye et al., (2010)			26.3 (use disorder), *263*[§]
Yee et al., (2022)	92.2, *95*[§]		
Zimmermann et al., (2012)	25.0, *30*[$$]		10.0 (dependence) – 30.8 (abuse), *12–37*[$$]

[$] *Includes problematic use up to disorder level: harmful, misuse, abuse, dependence, or disorder level*

[%] *Includes assessment periods up to 12 months prior to treatment entry: current, 1-, 3-, 6-, or 12-months*

[§] *Assessment period "current"; i.e., at admission to treatment or undefined*

[†] *Assessment period 1 month prior to treatment entry*

[‡] *Assessment period 6 months prior to treatment entry*

[#] *Assessment period 12 months prior to treatment entry*

[$$] *Assessment period 3 months prior to treatment entry*

Supplementary Table 6: Prevalence of co-occurring tobacco use among people accessing mental health treatment

Study	Any use		Problematic use[$]	
	Lifetime	Past 12 months[%]	Lifetime	Past 12 months[%]
**Tobacco %, *n***				
Azraai et al., (2021)		47.6, *232*[§]		
Bartlem et al., (2015)		50.7, *283*[§]		
Bartlem et al., (2018)		61.7, *1,277*[§]		
Davidson et al., (2001)		61.9, *145*[#]		
Fowler et al., (1998)		74.2, *144*[$$]		39.7 (more than 40 cigarettes/day), *77*[$$]
John et al., (2009)		64.0, *130*[§]		
Kavanagh et al., (2011)		76.3, *498*[†]		
Korman et al., (2023)		47.0, *47* [§]		
Lee et al., (2013)		71.9, *23*[§]		
Maccallum & Blaszczynski, (2002)		65.3, *49*[#]		37.3 (dependence), *28*[#]
Manning et al., (2017)		49.3, *413*[#]		40.7 (dependence), *341*[#]
Morgan et al., (2012)		66.1, *1206*[§]		
Wade et al., (2005)			77.0 (daily use), *97*	76.2 (daily use), *96*[#]
Zimmermann et al., (2012)		70.8, *85*[†]		16.7 (dependence) – 56.7 (abuse), *20–68*[†]

[$] *Includes problematic use up to disorder level: harmful, misuse, abuse, dependence, or disorder level*

[%] *Includes assessment periods up to 12 months prior to treatment entry: current, 1-, 3-, 6-, or 12-months*

[§] *Assessment period "current"; i.e., at admission to treatment or undefined*

[†] *Assessment period 3 months prior to treatment entry*

[#] *Assessment period 12 months prior to treatment entry*

[$$] *Assessment period 6 months prior to treatment entry*

Supplementary Table 7: Prevalence of co-occurring stimulant use among people accessing mental health treatment

Study	Any use		Problematic use[$]	
	**Lifetime**	**Past 12 months[%]**	**Lifetime**	**Past 12 months[%]**
**Any stimulants %, *n***				
Cleary et al., (2008)		14.4, *75*[§]		
Ogloff et al., (2004)			8.2 (abuse) – 17.8 (dependence), *6–13*	4.1 (abuse), *3*[†]
Ogloff et al., (2015)			27.7 (use disorder), *36*	3.8 (use disorder), *5*[†]
Sara et al., (2014)				13.9 (use disorder), *1,897*[†]
Suomi et al., (2014)		7.5, *15*[‡]		
Wye et al., (2010)				14.7 (use disorder), *147*[†]
**Amphetamines %, *n***				
Abrahams et al., (1970)		1.6, *16*[†]		1.8 (dependence), *18*[†]
Fowler et al., (1998)	34.0, *66*	9.8, *19*[#]	4.1 (abuse) – 9.3 (dependence), *8 – 18*	1.0 (abuse), *2*<br>1.0 (dependence), *2*[#]
Hides et al., (2007)				19.0 (dependence), *29*[$$]
John et al., (2009)		24.1, *49*[†]		
Kavanagh et al., (2011)		23.0, *150*[‡]		
Kent et al., (1995)			22.9 (abuse), *8*	
Lacey et al., (2007)				41.7 (injecting weekly), *39*[†]
Maccallum & Blaszczynski, (2002)		1.3, *1*[$$]		1.3 (abuse), *1*[$$]
Manning et al., (2017)		13.0, *109*[$$]		
Parker et al., (2005)		17.7, *50*[$$]		
Vaddadi et al., (1997)		39.6, *40*[†]		
Wade et al., (2005)			18.3 (use disorder), *23*	17.5 (use disorder), *22*[$$]

Yee et al., (2022)		91.3, *94*[†]		
Zimmermann et al., (2012)	54.2, *65*	6.7, *8*[‡]		1.7 (dependence) – 13.3 (abuse), *2–16*[‡]
**Cocaine %, *n***				
Fowler et al., (1998)	15.5, *30*	0, *0*[#]	0.0 (abuse) – 1.5 (dependence), *0-3*	0 (abuse, dependence), *0*[#]
Kavanagh et al., (2011)		1.9, *10*[‡]		
Ogloff et al., (2004)			4.1 (abuse), 4.1 (dependence), *3, 3*	1.3 (abuse), *1* [†]
Ogloff et al., (2015)			2.3 (use disorder), *3*	0 (use disorder), *0*[†]
Parker et al., (2005)		10.6, *30*[$$]		
Zimmermann et al., (2012)				0 (dependence) – 4.2 (abuse), *0–5*[‡]
**Ecstasy %, *n***				
Cleary et al., (2008)		6.2, *32*[§]		
Maccallum & Blaszczynski, (2002)		1.3, *1*[$$]		
Parker et al., (2005)		13.5, *38*[$$]		

[$] *Includes problematic use up to disorder level: harmful, misuse, abuse, dependence, or disorder level*

[%] *Includes assessment periods up to 12 months prior to treatment entry: current, 1-, 3-, 6-, or 12-months*

[§] *Assessment period 1 month prior to treatment entry*

[†] *Assessment period "current"; i.e., at admission to treatment or undefined*

[‡] *Assessment period 3 months prior to treatment entry*

[#] *Assessment period 6 months prior to treatment entry*

[$$] *Assessment period 12 months prior to treatment entry*

Supplementary Table 8: Prevalence of co-occurring opioid use among people accessing mental health treatment

Study	Any use		Problematic use[$]	
	Lifetime	Past 12 months[%]	Lifetime	Past 12 months[%]
**Any opioids %, *n***				
Fowler et al., (1998)	11.3 (non-prescribed) – 22.2 (prescribed, non-medical), *22–43*	2.6 (non-prescribed) – 5.1 (prescribed, non-medical), *5–10*[§]	0 (abuse) – 2.1 (dependence), *0–4* (prescribed, non-medical); 0.5 (abuse) – 2.6 (dependence), *1–5* (non-prescribed)	0 (dependence) – 0.5 (abuse), *0–1* (non-prescribed); 0 (abuse) – 1.5 (dependence), *0–3*[§] (prescribed, non-medical)
Hides et al., (2007)				3.3 (dependence), *5*[†]
John et al., (2009)				5.9 (misuse), *12*[‡]
Kavanagh et al., (2011)		6.7, *36*[#]		
Maccallum & Blaszczynski, (2002)				0 (use disorder), *0*[†]
Ogloff et al., (2004)			5.5 (abuse) – 13.7 (dependence), *4–10*	1.4 (abuse), *1*[‡]
Ogloff et al., (2015)			17.7 (use disorder), *23*	5.4 (use disorder), *7*[‡]
Wade et al., (2005)			12.7 (use disorder), *16*	11.1 (use disorder), *14*[†]
Zimmermann et al., (2012)		5.0, *6*[#]		0.8 (dependence) – 7.5 (abuse), *1–9*[#]
**Heroin %, *n***				
Cleary et al., (2008)		4.2, *22*[$$]		
Lacey et al., (2007)				28.3 (injecting weekly), *23*[‡]
Parker et al., (2005)		5.0, *14*[†]		

**Analgesic %, *n***		
Abrahams et al., (1970)	23.2, *233*[‡]	16.5 (dependence), *165*[‡]
Kavanagh et al., (2011)	5.4, *29*[#]	
**Morphine %, *n***		
Abrahams et al., (1970)		0.1 (dependence), *1*[‡]

[$] *Includes problematic use up to disorder level: harmful/risky/problematic/hazardous/chronic use, misuse, abuse, dependence, or disorder level*

[%] *Includes assessment periods up to 12 months prior to treatment entry: current, 1-, 3-, 6-, or 12-months*

[§] *Assessment period 6 months prior to treatment entry*

[†] *Assessment period 12 months prior to treatment entry*

[‡] *Assessment period "current"; i.e., at admission to treatment or undefined*

[#] *Assessment period 3 months prior to treatment entry*

[$$] *Assessment period 1 month prior to treatment entry*

Supplementary Table 9: Prevalence of co-occurring hallucinogen use among people accessing mental health treatment

Study	Any use		Problematic use[$]	
	Lifetime	Past 12 months[%]	Lifetime	Past 12 months[%]
**Any hallucinogens %, *n***				
Fowler et al., (1998)	37.1, *72*	3.1, *6*[§]	3.1 (abuse) – 4.1 (dependence), *6–8*	0 (abuse, dependence), *0*[§]
Kavanagh et al., (2011)		3.2, *17*[†]		
Kent et al., (1995)			20.0 (abuse), *7*	
Ogloff et al., (2004)			2.7 (dependence) – 5.5 (abuse), *2–4*	1.3 (abuse), *1*[‡]
Ogloff et al., (2015)			9.2 (use disorder), *12*	0.8 (use disorder), *1*[‡]
Sara et al., (2014)				1.0 (use disorder), *143*[‡]
Wade et al., (2005)			12.7 (use disorder), *16*	12.7 (use disorder), *16*[#]
Zimmermann et al., (2012)	37.5, *45*	2.5, *3*[†]		0 (dependence) – 4.2 (abuse), *0–5*[†]

[$] *Includes problematic use up to disorder level: harmful, misuse, abuse, dependence, or disorder level*

[%] *Includes assessment periods up to 12 months prior to treatment entry: current, 1-, 3-, 6-, or 12-months*

[§] *Assessment period 6 months prior to treatment entry*

[†] *Assessment period 3 months prior to treatment entry*

[‡] *Assessment period "current"; i.e., at admission to treatment or undefined*

[#] *Assessment period 12 months prior to treatment entry*

Supplementary Table 10: Prevalence of co-occurring inhalant use among people accessing mental health treatment

Study	Any use		Problematic use[$]	
	Lifetime	Past 12 months[%]	Lifetime	Past 12 months[%]
**Inhalant use %, *n***				
Fowler et al., (1998)	18.5, *36*	1.5, *3*[§]	0.5 (abuse) – 3.6 (dependence), *1–7*	0 (abuse, dependence), *0*[§]
Kavanagh et al., (2011)		1.5, *8*[†]		
Maccallum & Blaszczynski, (2002)				1.3 (abuse), *1*[‡]
Wade et al., (2005)			1.6 (use disorder), *2*	1.6 (use disorder), *2*[‡]
Zimmerman et al., (2012)		0.8, *1*[†]		0.0 (dependence) – 3.3 (abuse), *0–4*[†]

[$] *Includes problematic use up to disorder level: misuse, abuse, dependence, or disorder level*

[%] *Includes assessment periods up to 12 months prior to treatment entry: current, 1-, 3-, 6-, or 12-months*

[§] *Assessment period 6 months prior to treatment entry*

[†] *Assessment period 3 months prior to treatment entry*

[‡] *Assessment period 12 months prior to treatment entry*

Supplementary Table 11: Prevalence of co-occurring polydrug use among people accessing mental health treatment

Study	Any use		Problematic use[$]	
	Lifetime	Past 12 months[%]	Lifetime	Past 12 months[%]
**Polydrug use %, *n***				
Cleary et al., (2008)		20.8, *108*[§]		
Kavanagh et al., (2011)		22.8, *149*[†]		
Lambert et al., (2005)				16.4 (use disorder), *63*[†]
Maccallum & Blaszczynski, (2002)		2.7, *2*[‡]		
Reilly et al., (2019)				2.0 (use disorder), *42*[#]
Wade et al., (2005)			42.1 (use disorder), *53*	38.9 (use disorder), *49*[‡]

[$] *Includes problematic use up to disorder level: misuse, abuse, dependence, or disorder level*

[%] *Includes assessment periods up to 12 months prior to treatment entry: current, 1-, 3-, 6-, or 12-months*

[§] *Assessment period 1 month prior to treatment entry*

[†] *Assessment period 3 months prior to treatment entry*

[‡] *Assessment period 12 months prior to treatment entry*

Supplementary Table 12: PRISMA Checklist

Section and Topic	Item #	Checklist item	Location where item is reported
**TITLE**			
Title	1	Identify the report as a systematic review.	Title page
**ABSTRACT**			
Abstract	2	See the PRISMA 2020 for Abstracts checklist.	
**INTRODUCTION**			
Rationale	3	Describe the rationale for the review in the context of existing knowledge.	Pg 3-4
Objectives	4	Provide an explicit statement of the objective(s) or question(s) the review addresses.	Pg.4
**METHODS**			
Eligibility criteria	5	Specify the inclusion and exclusion criteria for the review and how studies were grouped for the syntheses.	Pg.5
Information sources	6	Specify all databases, registers, websites, organisations, reference lists and other sources searched or consulted to identify studies. Specify the date when each source was last searched or consulted.	Pg.5
Search strategy	7	Present the full search strategies for all databases, registers and websites, including any filters and limits used.	Supplementary Table 1
Selection process	8	Specify the methods used to decide whether a study met the inclusion criteria of the review, including how many reviewers screened each record and each report retrieved, whether they worked independently, and if applicable, details of automation tools used in the process.	Pg.5-6
Data collection process	9	Specify the methods used to collect data from reports, including how many reviewers collected data from each report, whether they worked independently, any processes for obtaining or confirming data from study investigators, and if applicable, details of automation tools used in the process.	Pg.6
Data items	10a	List and define all outcomes for which data were sought. Specify whether all results that were compatible with each outcome domain in each study were sought (e.g. for all measures, time points, analyses), and if not, the methods used to decide which results to collect.	Pg.6
	10b	List and define all other variables for which data were sought (e.g. participant and intervention characteristics, funding sources). Describe any assumptions made about any missing or unclear information.	Pg.6
Study risk of bias assessment	11	Specify the methods used to assess risk of bias in the included studies, including details of the tool(s) used, how many reviewers assessed each study and whether they worked independently, and if applicable, details of automation tools used in the process.	Pg.6-7
Effect measures	12	Specify for each outcome the effect measure(s) (e.g. risk ratio, mean difference) used in the synthesis or presentation of results.	Pg.7
Synthesis methods	13a	Describe the processes used to decide which studies were eligible for each synthesis (e.g. tabulating the study intervention characteristics and comparing against the planned groups for each synthesis (item #5)).	Pg.7
	13b	Describe any methods required to prepare the data for presentation or synthesis, such as handling of missing summary statistics, or data conversions.	Pg.6-7
	13c	Describe any methods used to tabulate or visually display results of individual studies and syntheses.	NA

Section and Topic	Item #	Checklist item	Location where item is reported
	13d	Describe any methods used to synthesize results and provide a rationale for the choice(s). If meta-analysis was performed, describe the model(s), method(s) to identify the presence and extent of statistical heterogeneity, and software package(s) used.	Pg.7
	13e	Describe any methods used to explore possible causes of heterogeneity among study results (e.g. subgroup analysis, meta-regression).	Pg.7
	13f	Describe any sensitivity analyses conducted to assess robustness of the synthesized results.	Pg.7
Reporting bias assessment	14	Describe any methods used to assess risk of bias due to missing results in a synthesis (arising from reporting biases).	NA
Certainty assessment	15	Describe any methods used to assess certainty (or confidence) in the body of evidence for an outcome.	NA
**RESULTS**			
Study selection	16a	Describe the results of the search and selection process, from the number of records identified in the search to the number of studies included in the review, ideally using a flow diagram.	Pg.7-8
	16b	Cite studies that might appear to meet the inclusion criteria, but which were excluded, and explain why they were excluded.	Pg.7-8
Study characteristics	17	Cite each included study and present its characteristics.	Supplementary Table 1
Risk of bias in studies	18	Present assessments of risk of bias for each included study.	Supplementary Table 1
Results of individual studies	19	For all outcomes, present, for each study: (a) summary statistics for each group (where appropriate) and (b) an effect estimate and its precision (e.g. confidence/credible interval), ideally using structured tables or plots.	Supplementary Tables
Results of syntheses	20a	For each synthesis, briefly summarise the characteristics and risk of bias among contributing studies.	Supplementary Table 1
	20b	Present results of all statistical syntheses conducted. If meta-analysis was done, present for each the summary estimate and its precision (e.g. confidence/credible interval) and measures of statistical heterogeneity. If comparing groups, describe the direction of the effect.	Figures, Pg. 10-17
	20c	Present results of all investigations of possible causes of heterogeneity among study results.	Pg.10-18
	20d	Present results of all sensitivity analyses conducted to assess the robustness of the synthesized results.	NA
Reporting biases	21	Present assessments of risk of bias due to missing results (arising from reporting biases) for each synthesis assessed.	NA
Certainty of evidence	22	Present assessments of certainty (or confidence) in the body of evidence for each outcome assessed.	NA
**DISCUSSION**			
Discussion	23a	Provide a general interpretation of the results in the context of other evidence.	Pg.18-27
	23b	Discuss any limitations of the evidence included in the review.	Pg.26
	23c	Discuss any limitations of the review processes used.	Pg.26

Section and Topic	Item #	Checklist item	Location where item is reported
	23d	Discuss implications of the results for practice, policy, and future research.	Pg.26-27
**OTHER INFORMATION**			
Registration and protocol	24a	Provide registration information for the review, including register name and registration number, or state that the review was not registered.	Pg.4-5
	24b	Indicate where the review protocol can be accessed, or state that a protocol was not prepared.	Pg.5
	24c	Describe and explain any amendments to information provided at registration or in the protocol.	NA
Support	25	Describe sources of financial or non-financial support for the review, and the role of the funders or sponsors in the review.	Cover page
Competing interests	26	Declare any competing interests of review authors.	Cover page
Availability of data, code and other materials	27	Report which of the following are publicly available and where they can be found: template data collection forms; data extracted from included studies; data used for all analyses; analytic code; any other materials used in the review.	NA

From: Page MJ, McKenzie JE, Bossuyt PM, Boutron I, Hoffmann TC, Mulrow CD, et al. The PRISMA 2020 statement: an updated guideline for reporting systematic reviews. BMJ 2021;372:n71. doi: 10.1136/bmj.n71
